# Sex of central neurons controls intestinal niche signalling to drive sex differences in stem cell activity and cancer susceptibility

**DOI:** 10.1038/s41467-026-76998-z

**Published:** 2026-08-22

**Authors:** Charlène Clot, Rénald Delanoue, Laura Helena Lavocat, Sélia Namouchi, Chloé Hérault, Bruno Hudry

**Affiliations:** Institut de Biologie Valrose, Université Côte d’Azur, CNRS, Inserm, Nice, France

**Keywords:** Intestinal stem cells, Gastric cancer, Sexual dimorphism

## Abstract

Sex differences in cancers arising in non-reproductive organs are widespread. While sex hormones and the intrinsic sexual identity of cancer cells are well-established contributors, it is unknown whether other mechanisms are involved. Here, we reveal an unexpected source of sex-biased cancer vulnerability in a *Drosophila* model of tumorigenesis. By combining genetically induced tumours with tissue- and cell type-specific sex reversals, we show that sex differences in oncogenesis can arise independently of both gonadal hormone signalling and the sexual identity of tumour cells themselves. Unexpectedly, we find that a sexually dimorphic neuronal circuit, classically associated with mating behaviour, is both necessary and sufficient to drive sex-biased intestinal tumorigenesis. The sex of these gut-innervating neurons controls tumour growth by stimulating local production of an insulin-like growth factor in the visceral muscles, a key component of the intestinal stem cell niche. Our findings reveal a previously unrecognised class of tumour-promoting input: the sex of central neurons can reprogram peripheral tumour-supportive environments. Under physiological conditions, this brain–gut pathway also modulates stem cell activity and organ size in a sex-specific manner to promote reproduction. Our work opens a new line of inquiry into how neuronal sex, particularly within the brain–gut axis, shapes physiology and disease, a field still predominantly studied in a male-only context.

## Abstract (web summary)

Sex hormones and cancer-cell sex influence tumor growth, but other sex-dependent drivers of oncogenesis remain unclear. Here, the authors reveal that the sex of central neurons alters the peripheral tumor-supportive niche to promote tumor growth.

## Introduction

The incidence of many cancers arising in non-reproductive organs differs significantly between men and women^1^. Overall, males experience a higher frequency of several cancer types—including brain, kidney, and liver cancers—resulting in nearly twice the mortality rate of females^2,3^. Notable exceptions exist, such as thyroid cancer, which is considerably more common in women: approximately 1 in 59 women will develop thyroid cancer, compared to just 1 in 160 men^1^. These sex biases in cancer incidence are also age-dependent. Age-stratified analyses reveal that premenopausal women have a higher risk than men for 13 different cancer types, with malignant melanoma showing the most pronounced female bias^4^.

Although differences in occupational exposure and lifestyle contribute to these disparities, intrinsic cellular and molecular mechanisms also play a fundamental role^2,3^. Among the most well-established biological drivers are gonadal steroid hormones, which influence cancer susceptibility through both intrinsic effects—such as regulation of cancer stem cell self-renewal^5–8^—and extrinsic effects, including modulation of the tumour microenvironment^9–11^. A well-characterised example of hormone-driven sex differences is hepatocellular carcinoma, which occurs more frequently in men^1^. Another major contributor to sex differences in cancer incidence and growth involves cell-intrinsic mechanisms driven by sex chromosome genes^12–15^. For instance, in a mouse model of colorectal cancer, the presence of the Y chromosome within tumour cells acts as a significant risk factor^12^. This effect is mediated by upregulation of a Y-linked histone demethylase, which promotes tumour progression^12^. As a result, male mice exhibit significantly poorer survival than females^12^, closely mirroring the sex disparities observed in human colorectal cancer^1^. While Y chromosome genes can also function as growth-inhibitors, X chromosome dosage represents another consistent tumour-suppressive mechanism, whereby genes that escape X inactivation provide a “double dose” of tumour suppressor activity in XX cells^16^. A well-established example is *Kdm6A*, part of the EXITS class (*Escape from X-Inactivation Tumour Suppressor genes*). *Kdm6A* encodes a histone H3 lysine 27 (H3K27) demethylase with tumour-suppressive roles across multiple cancer types, including myeloid leukaemia^17–19^, pancreatic^20,21^, bladder^22^, and metastatic lung cancers^23^. Its biallelic expression in XX cells confers enhanced protection, exemplifying the “double-dose tumour suppressor” effect.

Since the discovery of the first tumour suppressor gene in flies—*lethal giant larvae*—by Gateff and Schneiderman in 1967^24^, cancer research using model organisms such as *Drosophila melanogaster* has continued to thrive, owing to the fly’s remarkable genetic tractability^25^. Even in an era dominated by organoids and organs-on-a-chip, *Drosophila* is a powerful system for dissecting tumour–host interactions in vivo^26^. In addition to spontaneous tumour models^27,28^, specific cancer types—including intestinal cancers^29–31^—have been recreated in flies using targeted genetic manipulations^32^. These models have provided critical insights into diverse aspects of tumorigenesis, particularly those involving inter-organ signalling, such as metastasis^33,34^, cachexia^35–39^, and the impact of diet^40,41^. Yet, one important dimension remains largely unexplored in these systems —with few exceptions^42,43^: the mechanistic basis of sex differences in cancer biology.

**Fig. 1:**
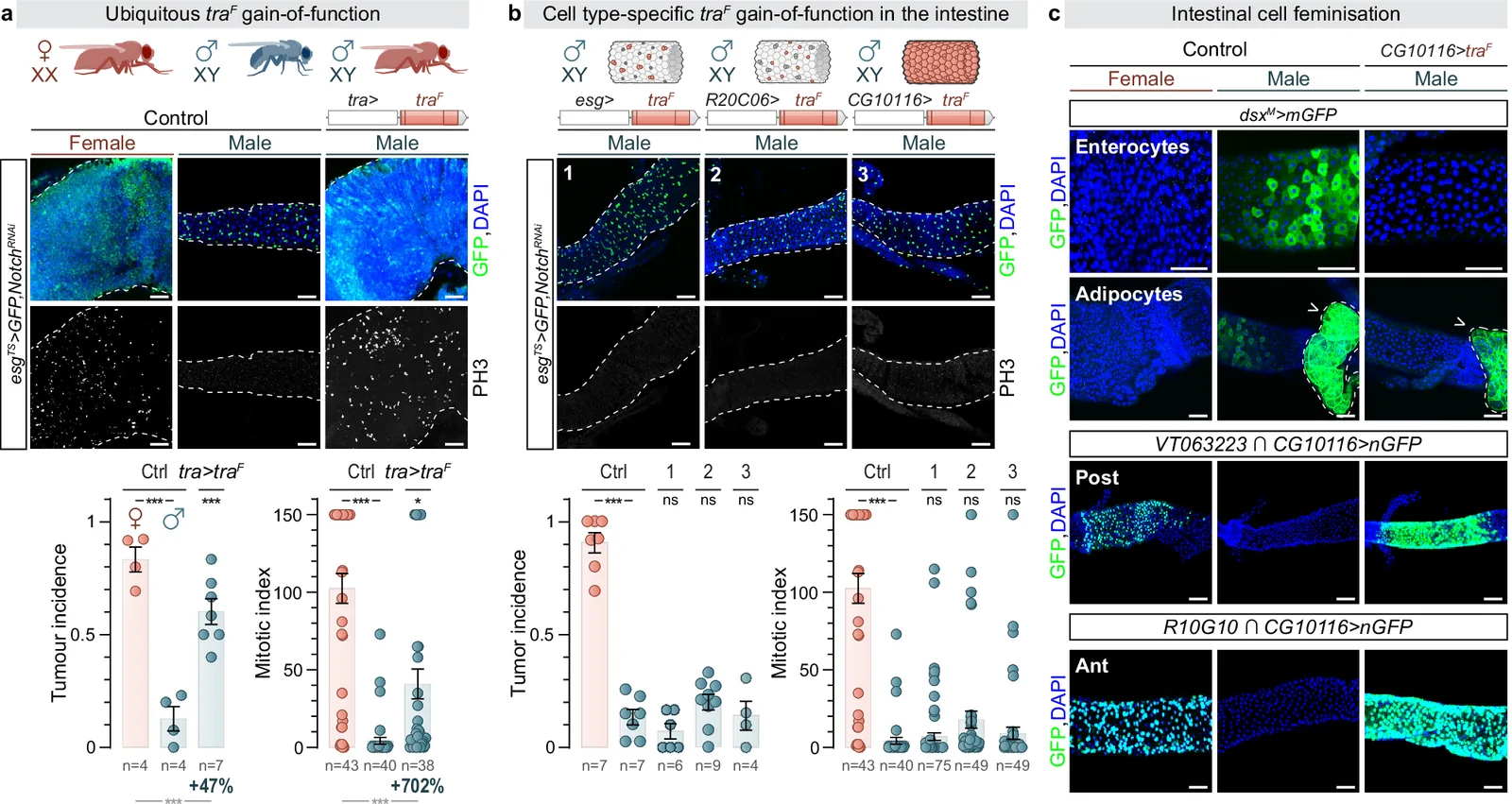
Sex-biased intestinal tumorigenesis is independent of gonadal hormones and tumour cell sexual identity. **a** Quantification of tumour incidence and neoplastic growth (pH3⁺ cell number) in adult midguts upon *Notch* silencing in progenitors, with or without ubiquitous *tra*^*F*^ expression in males. Confocal images show tumour coverage in the R4 region (DAPI: DNA, blue; GFP: progenitors, green; pH3: dividing cells, grey). *p  =  0.0187, ***p  <  0.0002 (one-way ANOVA). **b** Tumour incidence and neoplastic growth in adult male intestines following *tra*^*F*^ expression in progenitors (*esg*), enteroendocrine cells (*R20C06*), or all epithelial cells (*CG10116*). Confocal images as in (**a**). ***p  <  0.0001 (one-way ANOVA). **c** Expression of *dsx*^*M-LexA*^, *VT063223* ∩ *CG10116*, and *R10G10* ∩ *CG10116* reporters in the anterior (Ant) or posterior (Post) region of control and *tra*^*F*^-expressing male intestines (*CG10116>tra*^*F*^) (DAPI: DNA, blue; reporter-positive cells: GFP, green). Expression of *dsx*^*M-LexA*^ reporter in adipocytes (arrowheads) associated with the posterior midgut. In all panels, females are shown in red and males in blue. For tumour incidence, n=number of experiments; for mitotic index, n=number of midguts. Asterisks indicating statistically significant differences are shown in black at the top of the graphs for comparisons within sexes (and between female and male controls), and in grey at the bottom for comparisons between sexes (non-significant (ns): *p* > 0.05; ∗: 0.05 > *p* > 0.01; ∗∗: 0.01 > *p* > 0.001; ∗∗∗*p* < 0.001). Data from ≥3 independent experiments; bars represent mean ± SEM. Scale bars: 50 µm.

Interestingly, a recent study found a striking female bias in a *Drosophila* model of intestinal tumours^44^. In this model, *Notch* (*N*) is specifically silenced in adult intestinal progenitors, where it is essential for commitment to the enterocyte (EC) fate^29,30^. Loss of *N* function blocks differentiation, leading to the accumulation of immature cells that form small clusters of differentiation-defective precursors^45–47^. These clusters expand via autocrine mitogenic signalling^45^. Tumours consistently originate in the posterior midgut (the R4 region) and expand anteriorly, progressively disrupting epithelial architecture, displacing ECs, and inducing apoptosis^46^. Notably, anterior expansion is never observed without preceding hyperplasia in R4^48^. Strikingly, female flies are far more susceptible to tumour development in this model: two weeks after *N* inactivation, 83% of females develop *N*⁻ tumours, compared to only 13% of males (Fig. 1a and Supplementary Fig. 1a). Tumour growth also exhibits pronounced sex disparity, with female intestines containing an average of 102 mitotic neoplastic cells versus fewer than 4 in males (Fig. 1a and Supplementary Fig. 1a). This proliferative difference results in significantly larger tumours in females (Fig. 1a).

These striking sex differences, together with the well-characterised sex determination pathways in *Drosophila*, prompted us to investigate the cellular and molecular basis of this female-biased tumour susceptibility. In flies, as in mammals, females carry two X chromosomes, whereas males have one X and one Y^49,50^. This chromosomal complement determines cellular sexual identity via a splicing cascade that controls expression of the RNA-binding protein Transformer^F^ (Tra^F^), the master regulator of female identity, which is produced exclusively in XX cells^51^. Here, using the *N*-deficient tumour model, we find that sex-biased tumorigenesis in the gut is independent of gonadal hormones and of the sexual identity of the tumour cells themselves. Instead, we show that the sexual identity of central distant neurons regulates tumour expansion via insulin signalling. Specifically, we identify a local, female-enriched growth factor—Insulin-like peptide 3 (Ilp3)—that is secreted by the intestinal stem cell (ISC) niche, the visceral muscles, in response to the sexual identity of a defined population of gut-innervating neurons. This Ilp3 signal drives female-specific tumour growth. Notably, under physiological conditions, the same Ilp3-mediated pathway contributes to the regulation of gut size and reproductive capacity.

Together, our findings demonstrate that the intrinsic sexual identity of the brain–gut axis—independent of both gonadal hormones and tumour cell sex—governs sex-specific tumorigenesis. They reveal a critical and previously underappreciated role for cellular sex in a key component of the tumour microenvironment: central neurons. Under physiological conditions, this sex-dependent inter-organ signalling modulates adult stem cell activity and organ size, shaping female-biased gut physiology to support reproductive capacity.

## Results

### Sex-biased intestinal tumorigenesis is independent of gonadal hormones and tumour cell sexual identity

The demonstration of a female bias in an animal model of intestinal tumours^44^ prompted us to investigate the cellular and molecular basis of this sex-specific vulnerability. To begin dissecting the mechanisms underlying sex differences in oncogenesis, we first asked whether these disparities are controlled by genetic sex. To test this, we used a constitutively feminising allele of *tra*^*F*^—the female sex-determining gene—which we recently developed^51,52^. In this system, *tra*^*F*^ is ectopically expressed under the control of its own promoter in XY individuals, where it is normally not produced, thereby inducing somatic feminisation. This boosted tumour incidence in males by 47% and caused a sevenfold increase in neoplastic proliferation (Fig. 1a). Combined with previous findings that *tra*^*F*^ loss in XX flies abolishes sex differences^44^, these results demonstrate that Tra^F^ governs sex-specific susceptibility to intestinal tumour formation. Notably, Tra^F^-induced feminisation is effective in all tissues except the gonads: feminised XY flies fail to develop ovaries due to germline degeneration^53–55^, while *tra*^*F*^-null XX flies develop atrophic gonads and lack testes^53–55^. Thus, these manipulations, which induce germline degeneration through germline–soma sexual incompatibility, effectively rule out a role for germ cell–derived factors—whether tumour-suppressive signals from XY germ cells or pro-tumour signals from XX germ cells—in driving female-biased oncogenesis. More broadly, they argue against a significant contribution of gonadal signalling, including sex hormones^56^, in this context. This is particularly notable given that gonadal steroid hormones are among the best-established biological drivers of sex differences in cancer susceptibility in mammals and invertebrates^2,3,56^.

Another well-characterised mechanism underlying sex differences in cancer involves the intrinsic influence of sex-linked genes within cancer cells themselves^12–15,42^. Expressing *tra*^*F*^ specifically in intestinal progenitors using the *esgarcot (esg)* driver failed to alter tumour incidence or proliferation in males (Fig. 1b), suggesting that tumour expansion requires *tra*^*F*^ activity outside of tumour cells. *N⁻* tumours comprise proliferative *esg*-positive cells and non-dividing Prospero-positive enteroendocrine (EE)-like cells^45–47^. To assess whether EE cells influence sex bias via paracrine or systemic signals, we either feminised them using the EE-specific enhancer *R20C06*^57^ (Fig. 1b) or ablated them by knocking down *scute* (*sc*) (Supplementary Fig. 1b), required for EE fate^46^. Neither manipulation affects sex differences in tumorigenesis (Fig. 1b). We then used the pan-midgut enhancer of *CG10116*^58^ to feminise the entire epithelium, including ECs. This also failed to promote tumour expansion in males (Fig. 1b). To verify the efficiency of our sex-reversal approach, we developed a new reporter for the male isoform of *doublesex* (*dsx*^*M-LexA*^) (Fig. 1c), the main effector of the sex determination pathway and direct target of Tra^F^, and isolated two female-specific intestinal enhancers^58^ (*R10G10* for *Myc*, *VT063223* for *BarH1*) (Fig. 1c). In *tra*^*F*^-expressing males, *dsx*^*M*^ expression is lost, and *R10G10* and *VT063223* are activated (Fig. 1c), confirming efficient feminisation of the intestinal epithelium. In contrast, *dsx*^*M*^ expression in adipocytes from the same individuals remains unchanged (Fig. 1c), demonstrating the strict tissue specificity of this gut feminisation line.

Collectively, these findings indicate that sex differences in intestinal oncogenesis arise independently of both gonadal hormone signalling and the intrinsic sexual identity of tumour or intestinal cells.

### Sexual identity of non-gut cells orchestrates insulin-dependent tumour expansion in the intestine

Our results indicate that female-biased tumour susceptibility is driven by *tra*^*F*^ expression in an as-yet unidentified non-tumour, non-intestinal somatic cell type. We hypothesised that the sexual identity of these cells triggers, via inter-organ communication, the activation of a signalling pathway in gut progenitors in a sexually dimorphic manner, leading to enhanced tumour expansion in females.

**Fig. 2:**
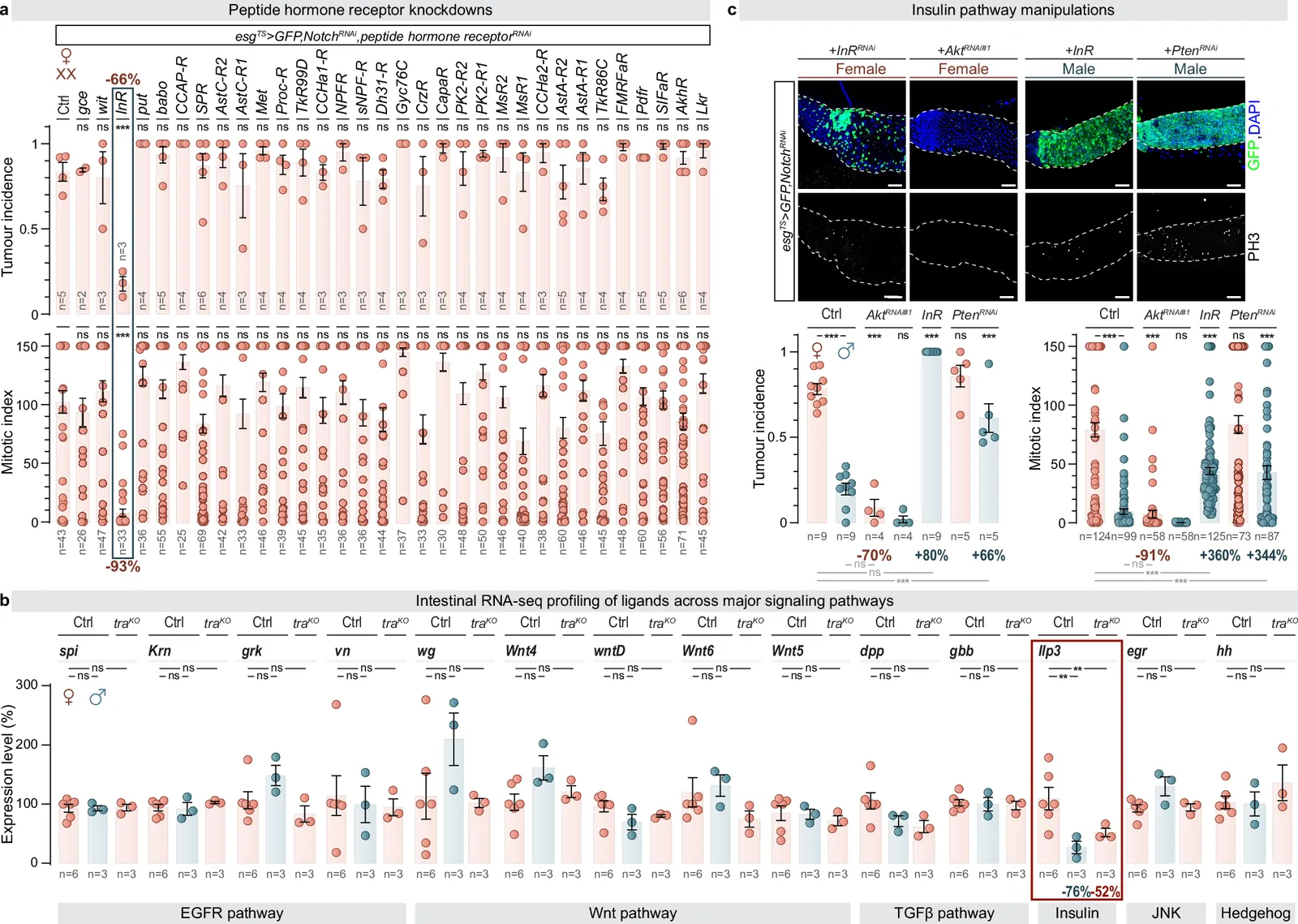
Sexual identity of non- intestinal cells drives insulin-dependent tumour expansion. **a** Tumour incidence and neoplastic growth (pH3⁺ cell number) in female midguts upon progenitor-specific *Notch* silencing combined with individual peptide hormone receptor knockdowns. ***p  <  0.0001 (one-way ANOVA). **b** Normalised expression levels of all known niche-derived ligands regulating intestinal stem cell proliferation across the EGFR, BMP/TGFβ, Wnt, JNK, Hedgehog, and Insulin pathways in the adult intestine of control females, males, and *tra*^*KO*^ females, based on transcriptional datasets from^44^ (GSE74772, GSE74774, one-way ANOVA without adjustment). **c** Tumour incidence and neoplastic growth in adult midguts of both sexes following *InR* silencing, *Akt* silencing, *InR* overexpression, or *Pten* knock-down in progenitors. Confocal images show tumour coverage in the R4 region (DAPI: DNA, blue; GFP: progenitors, green; pH3: dividing cells, grey). ***p  <  0.0001, **p  <  0.002 (one-way ANOVA). Scale bars: 50 µm. For tumour incidence and panel **b**, n=number of experiments; for mitotic index, n=number of midguts. Error bars represent mean ± SEM.

To identify this pathway, we conducted a targeted genetic screen by knocking down peptide hormone receptors in *esg*-expressing cells, focusing on major hormonal systems—like juvenile hormone signalling^59^—and neuropeptide receptors (Fig. 2a and Supplementary Fig. 2a). Strikingly, only disruption of the insulin receptor (*InR*) abolishes the female bias in intestinal tumorigenesis. In females, *InR* knockdown leads to a 66% reduction in tumour incidence and a fourteenfold decrease in neoplastic proliferation, both reduced to male-like levels (Fig. 2a and Supplementary Fig. 2a). To complement our functional approach and unbiasedly identify factors with sex-biased expression that may regulate ISC proliferation, we re-analysed two independent bulk RNA-seq datasets from adult midguts: (i) males versus females, and (ii) wild-type females versus masculinised females (ubiquitous *tra*^*F*^ loss-of-function). We systematically assessed all known niche-derived ligands involved in ISC regulation across the EGFR, BMP/TGFβ, Wnt, JNK, Hedgehog, and insulin pathways. Strikingly, none of the EGFR (*spi, krn, grk, vn*), BMP (*gbb, dpp*), Wnt (*wg, wnt4, wntD, wnt6, wnt5*), JNK (*egr*), or Hedgehog (*hh*) ligands exhibited sex-biased expression in either comparison (Fig. 2b). In contrast, *Ilp3* expression was ~fourfold higher in female midguts, and this bias was Tra^F^-dependent (Fig. 2b). These independent transcriptomic analyses align with our functional data, demonstrating that sex differences in stem cell proliferation are specifically associated with female-enriched insulin signalling.

To confirm that insulin pathway activity drives female-biased tumour growth, we silenced *Akt*, a core kinase in the insulin signalling cascade^60–62^, using three independent *RNAi* lines targeting distinct exons. As observed with *InR* knockdown, this resulted in a complete reduction of neoplastic proliferation in females to male-like levels (Fig. 2c and Supplementary Fig. 2b). To test whether ectopic activation of insulin signalling in males could mimic this female-biased phenotype, we manipulated the pathway in male gut progenitors by: (1) overexpressing *InR* in *esg*-positive cells (Fig. 2c); (2) expressing a constitutively active form of *Phosphatidylinositol 3-kinase 92E* (*Pi3K*), which acts downstream of InR and upstream of Akt^63,64^ (Supplementary Fig. 2b); and (3) silencing *Pten*, a negative regulator of Pi3K^65–67^ (Fig. 2c). In all three cases, tumour expansion is significantly upregulated in male intestines, while we found no effect in females (Fig. 2c).

Together, these findings establish that female-biased tumorigenesis is driven by sex-specific activation of the insulin/IGF pathway in intestinal progenitors, downstream of Tra^F^-dependent inter-organ signalling.

### Ilp3 is a niche-derived, female-enriched driver of gut tumour expansion

We then set out to identify the upstream factor responsible for the elevated insulin pathway activity in female gut stem cells. In *Drosophila*, InR and its downstream signalling cascade are activated by ligands that deliver proliferative signals^68,69^. While flies possess a single InR, it is stimulated by multiple Ilps, each exhibiting distinct spatiotemporal expression patterns that confer specificity to pathway activation. In adults, the main sources of Ilps are: (1) the neurosecretory insulin-producing cells (IPCs), which secrete Ilp2, Ilp3, and Ilp5 into the haemolymph^68,69^, and (2) the visceral muscles, which produce Ilp3 locally^70^. *Ilp1*, *Ilp4* and *Ilp6* are very weakly expressed in adults^71–73^, whereas Ilp7 and Ilp8 do not signal through InR. Indeed, Ilp8 is a relaxin-like hormone acting via the distinct receptor Lgr3^74,75^, while Ilp7 signals through Lgr4 to regulate egg laying^76,77^. We therefore excluded these Ilps, as our phenotype is adult-specific and strictly InR-dependent (Fig. 2a).

**Fig. 3:**
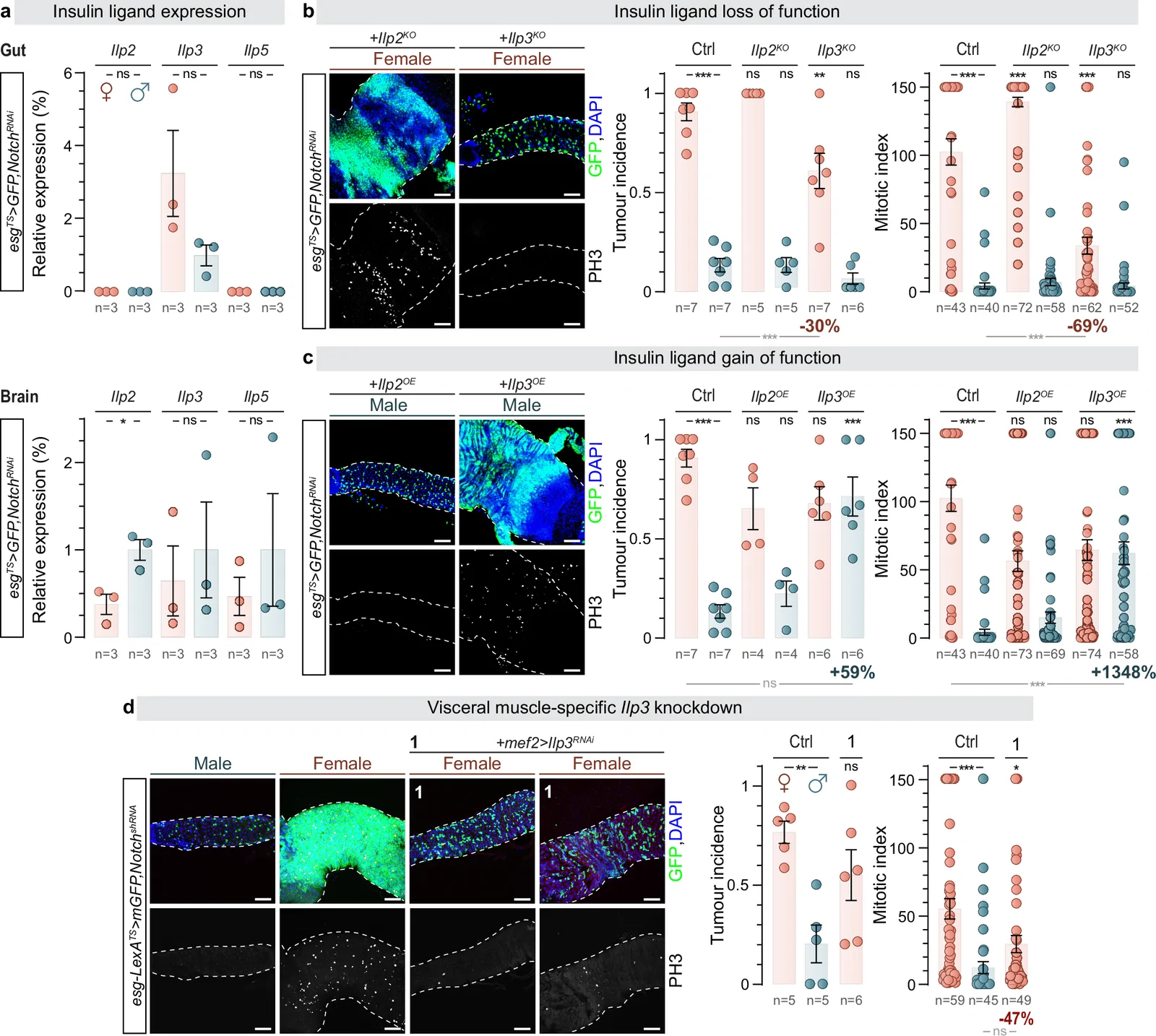
Ilp3 is a female-enriched, niche-derived driver of gut tumour growth. **a** RT-qPCR analysis of *Ilp2, Ilp3 and Ilp5* expression in adult midguts (top) and brains (bottom) of both sexes following progenitor-specific *Notch* silencing. *p  <  0.0243 (one-sided *t* test). **b** Tumour incidence and neoplastic growth (pH3⁺ cell number) in midguts with progenitor-specific *Notch* silencing combined with ubiquitous *Ilp2* and *Ilp3* knock-outs. ***p  <  0.0001 (one-way ANOVA). ***p  <  0.0001 (one-way ANOVA). **c** Tumour incidence and neoplastic growth in midguts of both sexes following progenitor-specific *Notch* knock-down with *Ilp2* or *Ilp3* overexpression. Confocal images as in (**b**). ***p  <  0.0006 (one-way ANOVA). **d** Tumour incidence and neoplastic growth in female midguts following progenitor-specific *Notch* knockdown combined with muscle-specific *Ilp3* knockdown. Confocal images show tumour coverage in the R4 region (DAPI: DNA, blue; GFP: progenitors, green; pH3: dividing cells, grey). ***p  <  0.0148, *p  <  0.002 (one-way ANOVA). Scale bars: 50 µm. For tumour incidence and panel **a**, n=number of experiments; for mitotic index, n=number of midguts. Error bars represent mean ± SEM.

We hypothesised that the sex-specific activation of InR signalling in gut progenitors is driven by a female-biased expression of at least one Ilp, thereby promoting enhanced tumour growth in females, since none of the core components of the insulin signalling pathway show sex-biased expression in the gut. Indeed, expression levels of the insulin receptor *InR*, its sole substrate *IRS* (which links activated InR to downstream signalling), downstream kinases (*PI3K*, *Pdk1* and *Akt*), and the negative regulator *Pten* are indistinguishable between males and females, as well as between wild-type and masculinised females (Supplementary Fig. 3a). To test this, we examined *Ilp* expression by RT-qPCR. In a tumoral context, none of the *Ilps* (*Ilp2*, *Ilp3*, *Ilp5*) display female-enriched expression in the brain (Fig. 3a). However, *Ilp3* expression is higher in female guts, while *Ilp2* and *Ilp5* are undetectable (Fig. 3a), consistent with our transcriptomic data showing a Tra^F^-dependent fourfold increase in *Ilp3* expression in female midguts (Fig. 2b). These data suggest that elevated Ilp3 abundance in females underlies their heightened susceptibility to *N*-deficient ISC tumours. To assess the requirement for specific Ilps in tumour growth, we analysed neoplastic proliferation in *Ilp* loss-of-function mutants. In females, tumour cell proliferation is reduced by 69% in *Ilp3* mutants compared to controls (Fig. 3b), whereas loss of IPC-derived *Ilp2* (Fig. 3b) or *Ilp5* (Supplementary Fig. 3b) has no effect. This indicates that systemic Ilps are not major contributors, and that Ilp3 is specifically required for tumour growth in females. We next asked whether Ilp3 is also sufficient to promote tumorigenesis in males. Strikingly, ectopic expression of *Ilp3*—but not *Ilp2*—in intestinal progenitors induces a robust increase in tumour growth in males, fully abolishing the sex difference in tumour progression (Fig. 3c). To directly test the contribution of visceral muscle-derived Ilp3, we generated a new tumour model using the *LexA/LexAop* system by establishing a *LexAop-N*^*shRNA*^ line. This system robustly induces intestinal tumours that retain a strong sex bias, as expected (Fig. 3d). Combining this model with muscle-specific *Ilp3* knockdown via the *Gal4/UAS* system resulted in a ~ 50% reduction in tumour growth in females (Fig. 3d), reducing tumour size to levels no longer significantly different from control males.

Together, these findings demonstrate that a local sex bias in Ilp3 production by the visceral muscles, a component of the ISC niche, is both necessary and sufficient to drive neoplastic proliferation and establish sex differences in oncogenesis. Moreover, Ilps display functional specificity in this context and are not functionally interchangeable.

### Neuronal sex dictates sexual dimorphism in gut tumours

**Fig. 4:**
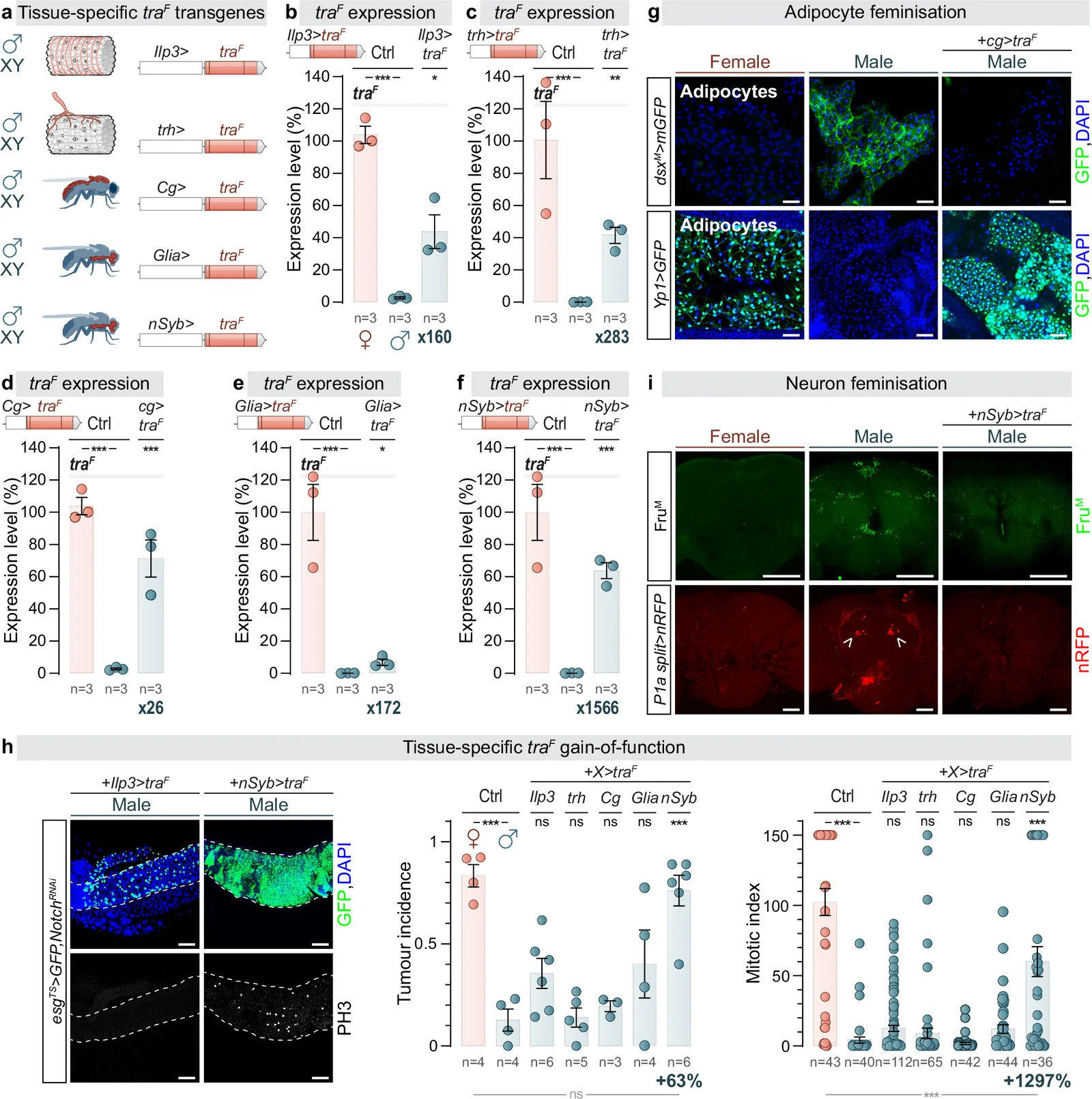
Neuronal sex dictates sexual dimorphism in gut tumours. **a** Tissue-specific feminisation via *tra*^*F*^ expression in *Ilp3*-expressing cells (*Ilp3*), tracheal cells (*trh*), adipose tissue and haemocytes (*Cg*), glia, or neurons (*nSyb*). RT–qPCR analysis of *tra*^*F*^ expression in control and *Ilp3>tra*^*F*^ (**b**), *trh>tra*^*F*^ (**c**), *Cg>tra*^*F*^ (**d**), *glia>tra*^*F*^ (**e**), and *nSyb>tra*^*F*^ (**f**) males. ***p  < 0.001, **p  <  0.0012, *p  < 0.023 (one-sided *t* test). **g** Expression of the Tra^F^>Dsx^F^ target *Yolk protein 1* (*Yp1*) and Tra^F^ target *dsx*^*M*^ in adipocytes of control and *Cg>tra*^*F*^ males (DAPI, blue; *Yp1* > *GFP*, green). **h** Tumour incidence and neoplastic growth in adult intestines following progenitor-specific *Notch* silencing combined with *tra*^*F*^ expression in *Ilp3*-expressing cells (*Ilp3*), tracheal cells (*trh*), adipose tissue and haemocytes (*Cg*), glia, or neurons (*nSyb*). Confocal images show tumour coverage in the R4 region (DAPI, blue; GFP, green; pH3, grey). ***p  <  0.0001 (one-way ANOVA). **i** Male-specific Fru^M^ protein expression in adult brains of control and *nSyb>tra*^*F*^ males (anti-Fru^M^, green). Visualisation of male-specific *fru*^*P1a*^ neurons (arrowheads) in brains of control and *nSyb>tra*^*F*^ males (*P1a split* > *nRFP*, red). Scale bars: 50 µm. For tumour incidence and RT–qPCR analysis, n=number of experiments; for mitotic index, n=number of midguts. Error bars represent mean ± SEM.

We next sought to determine where Tra^F^ is required to drive the female-biased expression of *Ilp3* in visceral muscles. The simplest hypothesis was that Tra^F^ acts within Ilp3-producing cells themselves. However, feminising male IPCs and visceral muscles by expressing *tra*^*F*^ under *Ilp3* regulatory elements (Fig. 4a, b) had no significant impact on tumour incidence or growth (Fig. 4h). This finding indicates that the key input controlling female-specific Ilp3 production lies outside of *Ilp3*-expressing cells.

To identify the relevant source tissue, we feminised specific cell types or organs by restricting *tra*^*F*^ gain-of-function to defined domains using tissue-specific enhancers (Fig. 4a). We first performed control experiments to validate effective feminisation of the targeted tissues. RT–qPCR confirmed expression of the female *tra*^*F*^ isoform in male tissues across all lines (Fig. 4b–f). We then conducted functional assays based on the canonical role of Tra^F^ in sex-specific splicing. In adipocytes, Tra^F^ expression switches splicing of its direct target *dsx* (Supplementary Fig. 4a) and induces the Dsx^F^ target *Yolk protein 1* (*Yp1)*^50,78^ (Supplementary Fig. 4a). In contrast, expression of the Dsx^F^ target *desatF*^79^ in oenocytes remains unchanged (Supplementary Fig. 4a), demonstrating strict tissue specificity. Consistently, our newly generated *dsx*^*M*^*-LexA* reporter shows complete loss of male identity in adipocytes (Fig. 4g), accompanied by active acquisition of female features, as indicated by *Yp1* induction (Fig. 4g), with no detectable effects in other tissues (Supplementary Fig. 4b). Despite successful feminisation, targeting tracheal cells (*trachealess* (*trh*)^80^-*tra*^*F*^, Supplementary Fig. 4c), adipocytes, immune cells (*Collagen type IV alpha 1* (*Cg*)^81^-*tra*^*F*^), or glia (*Glia*^82^-*tra*^*F*^) all fail to alter the female-biased tumour phenotype (Fig. 4h), suggesting that these tissues are not responsible for the sex-specific induction of intestinal *Ilp3* expression.

Finally, we assessed a potential neuronal contribution by expressing Tra^F^ in all mature neurons using the promoter of the pan-neuronal gene *n-Synaptobrevin*^83^ (*nSyb*-*tra*^*F*^). Full neuronal feminisation was confirmed by immunostaining, showing loss of the male-specific isoform of the Tra^F^ target Fruitless (Fru^M^) (Fig. 4i), elimination of male-specific neurons (Fig. 4i), and consequent male sterility (Supplementary Fig. 4d). Strikingly, feminising neurons leads to a 63% increase in tumour incidence, along with a thirteenfold rise in mitotic cells per tumour (Fig. 4h). We conclude that the sexual identity of neurons plays a critical role in driving sex-biased intestinal tumorigenesis.

### Sexual identity of *fruitless*^*P1*^ neurons governs intestinal tumour sex bias

We next focused on identifying the specific neuronal network responsible for regulating the female-biased tumour phenotype. The gastrointestinal tract functions in close coordination with other organs via neurons that innervate the gut, forming what is known as the brain–gut axis^84,85^. To examine involvement of this axis, we first targeted subpopulations of gut-innervating neurons described in the literature. These are broadly categorised into two classes: the first includes neurons projecting to the anterior gut, with cell bodies primarily in the brain (such as *Ilp2*^86^-, or *Dh44*^87^-expressing neurons); the second class innervates the posterior midgut and consists of neurons with cell bodies in the ventral nerve cord (such as Ilp7^86^-, Pdf^88^-, and ARCEN-positive neurons^89^). In addition, we identified and tested four novel populations (*R76F12*, *R37F03*, *R22C05*, and *R25F07*) that innervate the posterior midgut, as revealed by the K-Gut project^90^. Feminising each of these neuronal subsets by expressing *tra*^*F*^ failed to alter tumour incidence or size in male midguts (Supplementary Fig. 4e), indicating that none of them individually account for the sex-biased tumour growth.

**Fig. 5:**
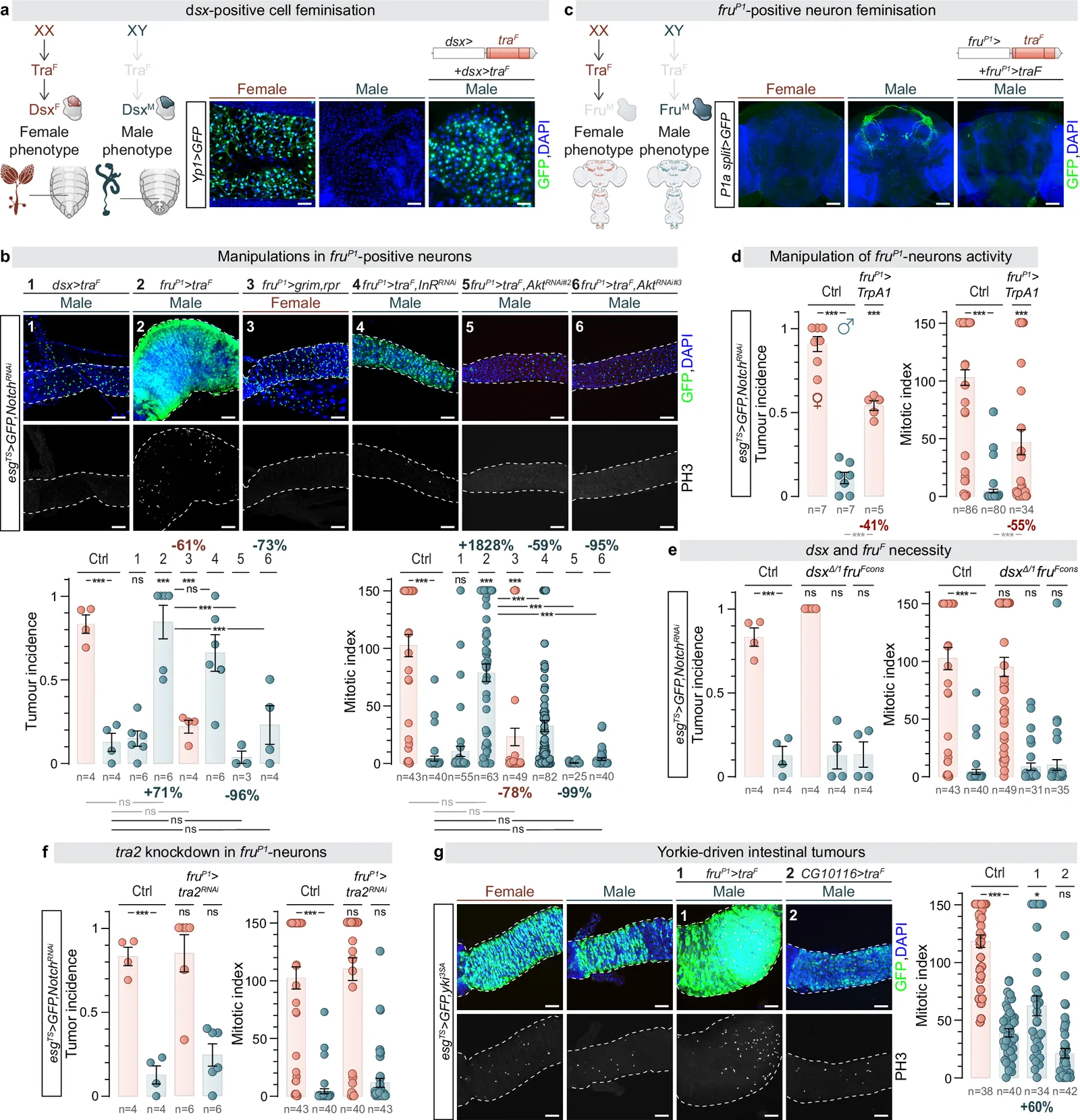
Sexual identity of *fruitless*^*P1*^ neurons governs intestinal tumour sex bias. **a** Expression of the Tra^F^>Dsx^F^ target *Yolk protein 1* (*Yp1*) in adipocytes of control and *dsx>tra*^*F*^ males (DAPI, blue; *Yp1* > *GFP*, green). **b** Tumour incidence and neoplastic growth following progenitor-specific *Notch* silencing under the indicated conditions: *tra*^*F*^ expression in *dsx*-expressing cells (*dsx>tra*^*F*^, condition 1); *fru*^*P1*^ neurons with (conditions 4–6: *fru*^*P1*^*>tra*^*F*^ + *InR*^*RNAi*^*, Akt*^*RNAi#2*^*, or Akt*^*RNAi#3*^) or without (condition 2: *fru*^*P1*^*>tra*^*F*^) *InR*/*Akt* knockdown in progenitors; and *fru*^*P1*^ neuron ablation (condition 3: *fru*^*P1*^*>grim,rpr*). Confocal images show tumour coverage in the R4 region (DAPI, blue; GFP, green; pH3, grey). **c** Visualisation of male-specific *fru*^*P1a*^ neurons in brains of control and *fru*^*P1*^>*tra*^*F*^ males (DAPI, blue; *P1a split > GFP*, green). Tumour incidence and neoplastic growth following progenitor-specific *Notch* silencing under the indicated conditions:  *fru*^*P1*^ neuronal activation (*fru*^*P1*^*>TrpA1*) (**d**); *dsx* mutant males (*dsx*^*Δ/1*^) or males expressing *fru*^*Fcons*^ (**e**); *tra2* knockdown in *fru*^*P1*^ neurons (**f**). Confocal images show tumour coverage in the R4 region (DAPI, blue; GFP, green; pH3, grey). **g** Neoplastic growth following progenitor-specific overexpression of a constitutive active from of *yorkie (yki*^*S3A*^*)* combined with *tra*^*F*^ expression in *fru*^*P1*^-neurons (*fru*^*P1*^*>tra*^*F*^, condition 1) or intestinal cells (*CG10116>tra*^*F*^, condition 2). Confocal images as in (**b**). ***p  <  0.0001, *p = 0.01 (one-way ANOVA). Scale bars: 50 µm. For tumour incidence, n=number of experiments; for mitotic index, n=number of midguts. Error bars represent mean ± SEM.

We then turned our attention to sexually dimorphic neurons, hypothesising that sex differences in intestinal tumorigenesis are governed by neural circuits that differ between males and females. In *Drosophila*, sexual dimorphism in the nervous system is largely attributed to two groups of neurons specified by the transcription factors Fru^91–94^ and Dsx^78,95,96^, both of which undergo sex-specific splicing directed by Tra^F^ ^97^. These circuits control nearly all known sex-specific behaviours^98–101^, including courtship^102–104^ and aggression^105^. To test their involvement in sex-biased tumour formation, we first expressed *tra*^*F*^ under the control of the *dsx* promoter in males (Fig. 5a). In addition to specifying sexually dimorphic neurons in the central and peripheral nervous systems, *dsx* is also expressed in non-neuronal tissues such as adipocytes^106^. Males expressing *tra*^*F*^ in *dsx*-positive cells ectopically express the female-specific gene *Yp1* in the fat body (Fig. 5a), confirming effective feminisation, but do not develop tumours (Fig. 5b, condition 1). Consistent with these results, *dsx*-null mutant flies exhibited female-biased intestinal tumorigenesis similar to wild-type controls (Fig. 5e), indicating that *dsx*-positive neurons are dispensable for this phenotype.

Finally, we turned to neurons expressing Fru, the other key regulator of sex-specific neuronal identity in the brain. We constitutively feminised *fru*-positive neurons by expressing *tra*^*F*^ under the control of the *fru*^*P1*^ promoter (Supplementary Fig. 5a). The *fru*^*P1*^ neuronal network includes sex-specific neurons present exclusively in one sex. This causes loss of the male-specific *fru* isoform, confirmed by RT-qPCR (Supplementary Fig. 5b) and immunostaining (Supplementary Fig. 5c). Expression of *tra*^*F*^ in these neurons eliminates male-specific neurons^107^ (Fig. 5c) and induces the emergence of female-specific neurons^108^ in males (Supplementary Fig. 5c). Furthermore, feminisation of *fru*^*P1*^ neurons abolishes the formation of a male-specific abdominal muscle required for copulatory bending^109^ (Supplementary Fig. 5d), resulting in male sterility (Supplementary Fig. 5e) without affecting sex differences outside the brain (Supplementary Fig. 5b). This validates the specificity and efficacy of our sex-reversal approach in this neuronal population. Remarkably, *fru*^*P1*^*>tra*^*F*^ males develop large *N*-deficient ISC tumours, exhibiting an eighteenfold increase in neoplastic proliferation—identical to tumours in control females (Fig. 5b, condition 2). These results demonstrate that feminising the *fru*^*P1*^ neuronal population is sufficient to drive female-like tumour growth in males. To further assess the functional importance of these neurons, we asked whether their ablation would suppress tumour development in females. We combined *N* knockdown in intestinal progenitors with expression of the pro-apoptotic genes *grim* and *reaper* (*rpr*)^110^ in *fru*^*P1*^-positive neurons. Strikingly, ablation of the *fru*^*P1*^ neurons reduces tumour growth in females to levels comparable to males (Fig. 5b, condition 3). We then simultaneously induced *N* silencing in intestinal progenitors and activated *fru*^*P1*^ neurons using the warmth-activated channel TrpA1. This resulted in a 41% reduction in tumour incidence and a 55% decrease in tumour cell proliferation in females (Fig. 5d). These findings provide independent support, through neuronal activity modulation, that *fru*^*P1*^ neurons are key drivers of sex differences in intestinal tumour growth. Interestingly, this tumour-promoting role of Tra^F^ in *fru*^*P1*^-positive neurons appears to be independent of *fru* itself, as forced expression of the female *fru* isoform in males has no impact on tumour development (Fig. 5e). We next knocked down *transformer 2* (*tra2*)—the essential Tra^F^ cofactor—specifically in *fru*^*P1*^ neurons. This manipulation did not alter female-biased tumour growth (Fig. 5f). Notably, *tra2* silencing, and thus neuronal masculinisation, was induced concurrently with tumour formation, as *fru*^*P1*^*-Gal4* is controlled by temperature-sensitive *Tub-Gal80* used to trigger adult-onset *N* knockdown. These results indicate that adult-specific masculinisation of *fru*^*P1*^ neurons does not affect female-biased tumour susceptibility. This is consistent with previous studies showing that *fru*^*P1*^-dependent sex differences are established during a critical pupal period^111–113^. We therefore infer that Tra^F^ acts during this developmental window to programme *fru*^*P1*^ neurons in XX females for their role in regulating intestinal tumour growth, establishing a stable state that cannot be overridden in adulthood.

To assess the generality of our findings, we analysed two additional, independent intestinal tumour models. We activated the Hippo pathway by expressing a constitutively active form of Yorkie (*yki*^*3SA*^)^114,115^, and induced stem cell hyperproliferation via Shavenbaby/ovo (*ovo*^*ACT*^) expression^116^, which acts downstream of Wnt and EGFR signalling in intestinal stem cells. Consistent with the *N* model, both systems exhibit robust and reproducible sex differences: *ovo*^*ACT*^-induced tumours contain eightfold more proliferating cells than in males (Supplementary Fig. 5f), while *yki*^*3SA*^-driven tumours show a threefold increase in dividing cells in females (Fig. 5g). To test whether the underlying mechanism is conserved, we combined *yki*^*3SA*^ tumours with targeted feminisation of either gut cells or *fru*^*P1*^ neurons. As in the *N* model, gut feminisation did not alter the sex bias (Fig. 5g, condition 2), supporting a non–cell-autonomous effect. In contrast, feminisation of *fru*^*P1*^ neurons increased tumour growth in males (Fig. 5g, condition 1), albeit to a lesser extent.

Together, these results across distinct oncogenic contexts demonstrate that the *fru*^*P1*^ neuronal network is both necessary and sufficient to drive sex-biased tumour growth, and reveal a previously unrecognised, *fru*-independent role for Tra^F^ in governing sex differences in this neural population.

### *fru*^*P1*^ neurons control female-biased tumour growth via *Ilp3* expression in visceral muscle

We next investigated the link between the *fru*^*P1*^ neuronal network and sex-biased tumorigenesis, hypothesising that this connection involves the regulation of sex differences in Ilp3 production by visceral muscles. To test this, we first silenced *InR* in intestinal progenitors in males with feminised *fru*^*P1*^ neurons, within a tumoral context. This leads to a 60% reduction in cancer cell proliferation (Fig. 5b, condition 4), indicating that the tumour-promoting effect of *fru*^*P1*^ neurons depends on insulin pathway activity in the intestinal progenitors. This was further confirmed by silencing the core insulin pathway kinase *Akt* using two independent validated *RNAi* lines. In both cases, tumour growth in feminised males was fully suppressed, returning to levels indistinguishable from control males (Fig. 5b, conditions 5 and 6). Together, these results provide clear causal evidence that sex differences in tumour growth are driven by sex-biased insulin signalling. Interestingly, males with feminised *fru*^*P1*^ neurons also increase *Ilp3* expression in the gut to levels observed in females (Supplementary Fig. 5g). These results suggest that Tra^F^ activity in *fru*^*P1*^ neurons drives sex-biased tumorigenesis by regulating *Ilp3* expression in visceral muscle.

**Fig. 6:**
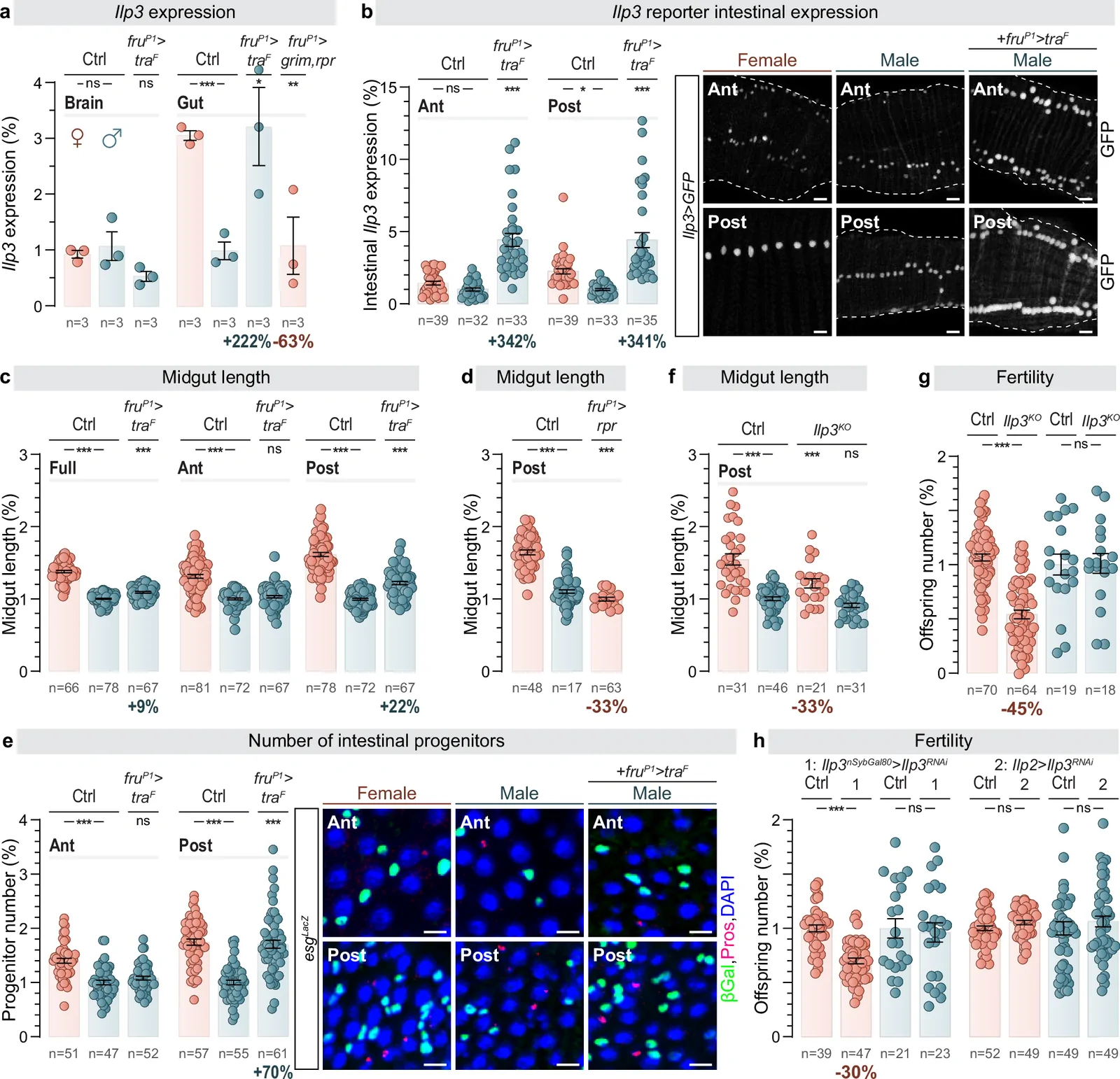
Sexual identity of *fru*^*P1*^ neurons orchestrates Ilp3-dependent regulation of gut size and fecundity. **a** RT-qPCR analysis of *Ilp3* expression in adult brains (left) and midguts (right) of controls, *fru*^*P1*^*>tra*^*F*^ males, and *fru*^*P1*^*>grim,rpr* females. *p  <  0.038, **p  <  0.0095, ***p  <  0.0001 (one-sided *t* test). **b** Visualisation of *Ilp3* expression in the anterior and posterior midgut regions of controls and *fru*^*P1*^*>tra*^*F*^ males (*Ilp3* > GFP, grey). *p  =  0.0117, ***p  <  0.0001 (one-way ANOVA). Scale bars: 50 µm. **c** Quantification of total and region-specific (anterior: Ant and posterior: Post) midgut length in controls and *fru*^*P1*^*>tra*^*F*^ males. ***p  <  0.0001 (one-way ANOVA). **d** Posterior midgut length quantification in controls and *fru*^*P1*^*>rpr fe*males. ***p  <  0.0001 (one-way ANOVA). **e** Quantification of progenitor number (*esg*-positive cells) in the anterior and posterior regions of controls and *fru*^*P1*^*>tra*^*F*^ males. Representative confocal images are shown (DAPI: DNA, blue; *esg-βGal*: progenitors, green; Pros: enteroendocrine cells, red). ***p  <  0.0001 (one-way ANOVA). Scale bars: 5 µm. n represents the number of fields quantified, with each condition including at least 10 independent animals. **f** Posterior midgut length quantification in control and *Ilp3* null mutant flies. ***p  <  0.0001 (one-way ANOVA). **g**, Fertility assay showing number of offspring in control and *Ilp3* null mutant flies. ***p  <  0.0001 (one-way ANOVA). **h** Fertility assay showing number of offspring in flies with *Ilp3* silencing in the visceral muscles only (*Ilp3*^*nSybGal80*^), or the insulin-producing cells (IPCs) only (*Ilp2* > ). ***p  <  0.0001 (one-way ANOVA). For panel **a**, n=number of experiments; for all the other panels, n=number of midguts, except in g and h n=number of individuals. Error bars represent mean ± SEM.

The *fru*^*P1*^ neuronal network is not known to participate in the brain–gut axis. To explore whether it directly connects to the intestine, we examined its anatomical projections. Strikingly, we find that specific subsets of *fru*^*P1*^ neurons densely innervate both the anterior and posterior regions of the midgut (Supplementary Fig. 6a). We next tested whether the sexual identity of *fru*^*P1*^ neurons regulates *Ilp3* expression in visceral muscles under physiological (non-tumoural) conditions. Using qPCR and immunostaining of dissected brains and guts, we find that while no *Ilp* genes display sex-biased expression in the brain (Supplementary Fig. 6b and c), *Ilp3* is expressed at threefold higher levels in the visceral muscles of females compared to males (Fig. 6a). Remarkably, feminising *fru*^*P1*^ neurons in males is sufficient to induce *Ilp3* expression in the gut at female levels (Fig. 6a), without altering expression in the brain. Conversely, genetic ablation of *fru*^*P1*^ neurons in females reduces *Ilp3* expression in the gut to male-like levels (Fig. 6a). To independently validate these findings, we combined an *Ilp3* transgenic driver line with a nuclear GFP^70,117^ (Fig. 6b and Supplementary Fig. 6d). This reporter confirmed that *Ilp3* is specifically expressed in visceral muscles, with a striking compartmentalisation along the anteroposterior axis. Expression is restricted to two domains: an anterior region (R1) and a posterior region (R4) (Supplementary Fig. 6d). Notably, only the posterior region—corresponding to the site of *fru*^*P1*^ neuronal innervation and the origin of *N*-deficient tumours—exhibits female-biased expression (Fig. 6b). Finally, combining the *Ilp3* reporter with ectopic *tra*^*F*^ expression in *fru*^*P1*^ neurons further confirmed that feminisation of this neural circuit in males induces *Ilp3* expression in the gut (Fig. 6b).

These findings establish that the sexual identity of *fru*^*P1*^ neurons controls sex-specific *Ilp3* expression in the intestine, uncovering a mechanistic pathway linking neuronal sexual identity to sex differences in intestinal tumour development.

### Sex-biased *Ilp3* expression from the niche controls gut size and fertility

As we observed that *Ilp3* expression in visceral muscles differs between males and females even under non-tumoral conditions, we next asked whether this sex-specific inter-organ communication between *fru*^*P1*^ neurons and the ISC niche contributes to physiological sex differences.

Since Ilp3 regulates ISC division rates^70^, we hypothesised that female-biased Ilp3 production influences organ size. Indeed, adult wild-type female midguts are approximately 40% longer than those of males (Fig. 6c), and silencing *tra*^*F*^ in intestinal progenitors reduces female gut length^44^. Conversely, forced expression of *tra*^*F*^ in male progenitors increases gut size (Supplementary Fig. 6e), indicating that the intrinsic sexual identity of gut cells contributes to sex differences in organ size. However, this accounts for only about one-third of the observed sexual dimorphism, suggesting that additional factors, external to the gut, are involved. To test whether the sexual identity of *fru*^*P1*^-expressing neurons contributes to this phenotype, we feminised these neurons in males. Strikingly, this significantly extend total midgut length (Fig. 6c). Further analysis using regional markers revealed that the posterior R4 region of the midgut, which corresponds to the domain innervated by *fru*^*P1*^ neurons, increases in size by 30%, while other intestinal regions remain unaffected (Fig. 6c and Supplementary Fig. 6f). As before, we performed the complementary experiment by ablating *fru*^*P1*^ neurons in females. This resulted in a 33% reduction in the size of the posterior midgut (Fig. 6d). Collectively, these findings demonstrate that *fru*^*P1*^ neuronal sexual identity exerts a greater influence on organ size than the intrinsic sex of gut cells, modulating gut length in a region-specific manner and playing a key role in establishing sex differences in intestinal morphology.

We next explored the cellular and molecular mechanisms mediating the effects of *fru*^*P1*^ neuronal sexual identity on gut size. Quantification of intestinal progenitors revealed a female-biased increase in progenitor number, which is abolished specifically in the posterior R4 region upon feminisation of *fru*^*P1*^ neurons in males (Fig. 6e). Indeed, *tra*^*F*^ expression in *fru*^*P1*^ neurons leads to a 70% increase in progenitor number specifically in the R4 domain of male guts (Fig. 6e and Supplementary Fig. 6g), indicating that the sexual identity of *fru*^*P1*^ neurons regulates ISC proliferation even under physiological conditions. Interestingly, this effect appears to be context-dependent. In a model of intestinal damage induced by dextran sodium sulphate (DSS), the female-biased proliferative response persisted regardless of *fru*^*P1*^ neuron sexual identity (Supplementary Fig. 6h) and is known to be driven by the intrinsic sex of the gut stem cells^44^. Thus, while *fru*^*P1*^ neurons modulate ISC proliferation in steady-state conditions, their influence is overridden in regenerative contexts.

At the molecular level, we investigated whether this Tra^F^-dependent inter-organ effect is functionally mediated by *Ilp3*, as observed in the tumour model. Strikingly, *Ilp3*-null mutant females display a 33% reduction in size of the posterior midgut (Fig. 6f), while mutant males are unaffected, indicating a female-specific requirement for *Ilp3* in gut size regulation. *Ilp3* is expressed in two adult tissues: the IPCs in the brain and the visceral muscles of the gut^117^. To determine which source is functionally relevant, we used two different *Ilp* driver lines: one expressed only in visceral muscles (*Ilp3*^*nSybGal80*^ > ), and another restricted to the IPCs (*Ilp2* > , Supplementary Fig. 6i). Using a validated *Ilp3* RNAi line (Supplementary Fig. 6j), we found that silencing *Ilp3* in muscles reduces female gut size by 30%, whereas knockdown in the IPCs alone has no effect (Supplementary Fig. 6i). Therefore, *Ilp3* expression in visceral muscles is responsible for female-biased gut size. Given that sexual dimorphism in physiology often supports sex-specific reproductive strategies shaped by anisogamy, we asked whether female-biased *Ilp3* expression contributes to female reproductive output. Indeed, remarkably, *Ilp3* mutant females exhibit a 45% reduction in offspring production, while male fecundity is unaffected (Fig. 6g). Consistent with our model, this function of Ilp3 is intestinally mediated: neuronal *Ilp3* knockdown has no effect (Fig. 6h), whereas visceral muscle-specific inhibition reduces female fertility by ~30% (Fig. 6h), with no detectable impact in males.

Together, our findings establish that the sexual identity of *fru*^*P1*^ neurons controls *Ilp3* expression in a regionally and sexually dimorphic manner, thereby regulating gut stem cell activity and organ size. This inter-organ communication pathway supports female-biased gut physiology that serves to maximise female reproductive capacity.

## Discussion

**Fig. 7:**
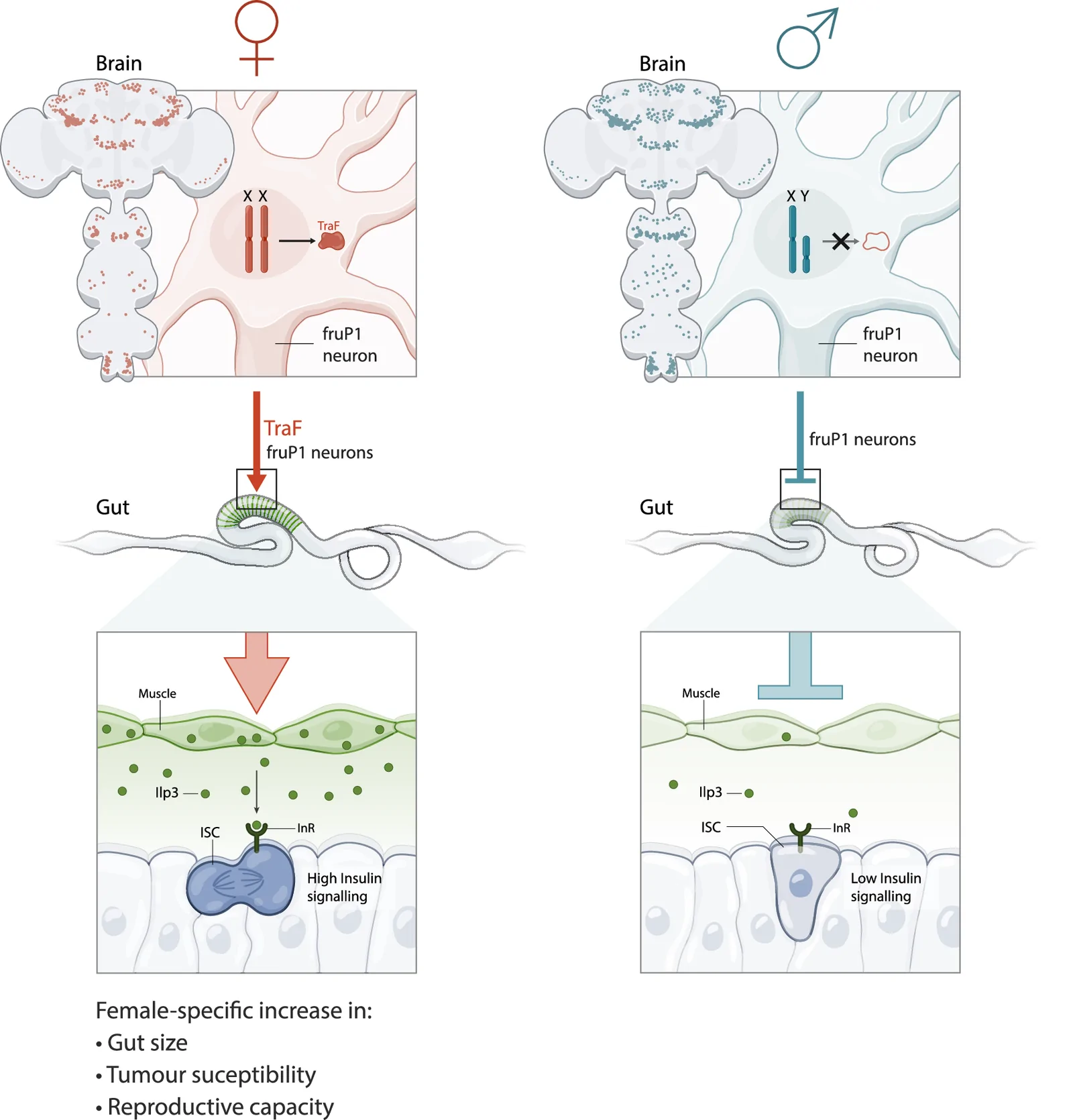
Sex of central *fru*^*P1*^ neurons programmes an intestinal niche that drives sex differences in stem cell activity and cancer susceptibility. The *fru*^*P1*^ sexually dimorphic neural circuit—previously known for regulating sexual orientation—is both necessary and sufficient to drive sex-biased intestinal tumorigenesis. The sex of these neurons controls tumour growth by stimulating local production of an insulin-like growth factor in the visceral muscles, a key component of the intestinal stem cell niche. Under physiological conditions, this brain–gut pathway modulates stem cell activity and organ size in a sex-specific manner to support reproduction.

Gonadal steroid hormones and sex chromosome-linked gene expression in oncogenic cells are well-established mechanisms by which biological sex shapes cancer biology. Here, we reveal a third mechanism: the impact of cellular sex in a non-tumour component of the microenvironment that promotes tumour growth via local induction of a mitogenic factor. This occurs in an unexpected niche compartment—central neurons. Two features distinguish this neural–tumour axis: (1) the cellular substrate—a sexually dimorphic neuronal circuit; and (2) the molecular effector—a niche-derived Ilp. Together, they define a previously unrecognised, neuronally driven mechanism by which cellular sex can modulate tumorigenesis and organ physiology (Fig. 7). *Drosophila* and human biology, as well as the specific features of their organ systems, are sufficiently distinct that our findings cannot be directly extrapolated to a specific human disease context, such as colorectal cancer. Nevertheless, the general principle that neuronal sexual identity can influence peripheral tumour niches may represent a conserved and previously unrecognized mechanism contributing to sex differences in tumour development across species.

We show that the *fru*^*P1*^ neuronal circuit, best known for directing male courtship^97,118^, also governs sex differences in the intestine. While *fru*^*P1*^-expressing neurons mediate sexual behaviours—including aggression^105,107,119,120^ and female reproductive behaviours^121–124^—our data indicate a broader biological strategy, coupling courtship and physiological sex differences through a shared neuronal substrate to optimise reproductive success. The downstream effectors of cellular sex in *fru*^*P1*^ neurons are unknown. We propose two models: (1) direct regulation of *Ilp3* expression in visceral muscle by *fru*^*P1*^-positive gut-innervating neurons, or (2) an indirect effect via a neuronal relay. Using synaptic markers, we find that *fru*^*P1*^ neurons form neuromuscular junctions selectively with circular visceral muscle cells (Supplementary Fig. 6k). As *Drosophila* neuromuscular junctions are glutamatergic^125–127^, we hypothesise that Ilp3 induction is driven by glutamatergic signalling. Notably, glutamate signalling is implicated in several human cancers^128,129^, including colorectal cancers^130,131^.

Most known tumour–neuron interactions involve peripheral neurons directly innervating tumour cells^129^. For example, serotonergic enteric neurons in mice promote tumour growth via stem cell activation^132^. In contrast, *fru*^*P1*^ neurons originate in the central nervous system and innervate the gut via long-range projections, representing a fundamentally distinct tumour-promoting input. In non-tumoural contexts, the best known peripheral target of *fru*^*P1*^ neurons are the sexually dimorphic muscles of Lawrence (MOL)^109^, whose male-specific development requires secretion of Activin-β by Fru-positive motoneurons^133^. While this process requires the male-specific Fru^M^ isoform^134,135^, our work uncovers a FruM-independent mechanism mediated by Tra^F^ in *fru*^*P1*^ neurons. Understanding how sex determinants modulate neural function in a cell-autonomous way will be key to deciphering sex differences in adult physiology. The widespread expression of Tra^F^ in the adult nervous system^51^—and of sex-linked genes in other species including human^136^—suggests this mechanism may be broadly relevant.

Crucially, we show for the first time that intrinsic sexual identity of neurons in the brain–gut axis has physiological consequences. This axis comprises diverse neuron types^84,85^ regulating gut function^84,87,137^ and adjacent organs (e.g., trachea^138^, Malpighian tubules^88^, as well as behaviours like feeding^139–141^ and defecation^142^). Despite this complexity, the role of neuronal sex in gut physiology has been largely overlooked. For instance, while glutamatergic motor neurons control hindgut contractions and sphincter activity^139^, and cholinergic enteric neurons promote gut regeneration^89^, these have been studied exclusively in one sex.

Our findings position *fru*^*P1*^ neurons as key drivers of intestinal sex differences, supporting an emerging model in which the adult gut epithelium is shaped by multiple, independent sex-differentiation pathways^44,56,59,143,144^. These include a gonad-derived peptide regulating sex-specific sugar metabolism^52^, and the intrinsic sex of gut stem cells controlling regeneration and organ size^44^. The mechanism we describe here—sexual identity of brain–gut neurons—acts in parallel to intrinsic gut sex, influencing organ size but not regenerative capacity. Whether it also contributes to other intestinal sex differences remains to be determined.

We further identify a previously unrecognised paracrine neural–tumour interaction, mediated through the visceral musculature. This sex-biased communication relies on spatially restricted production of Ilp3, a growth factor. While growth hormones are implicated in tumorigenesis in multiple organs (e.g., breast, prostate, colon), those cases involve autocrine growth factor production by cancer cells^145,146^. In contrast, we reveal a non-autonomous growth signal from a tissue outside the tumour, governed by neuronal sex.

A defining feature of this inter-organ signalling is distinct functional specificity of two Ilps: locally produced Ilp3, which activates stem cell proliferation, and circulating Ilp2, which does not. This non-redundancy may stem from differences in receptor affinity or signalling strength. Recent cryo-EM structures of InR bound to different Ilps^147^ provide a mechanistic framework for our findings. Each Ilp induces a distinct receptor conformation, with differing binding stoichiometries (for example, one Ilp2 versus three Ilp5 molecules per receptor complex). These differences translate into quantitative variation in receptor activation, with Ilp2 eliciting lower autophosphorylation than other Ilps, offering a possible molecular explanation for our observations. Alternatively, local modulators like Ilp-binding proteins could fine-tune ligand availability. One candidate is Ecdysone-inducible gene L2 (ImpL2), a factor expressed in intestinal progenitors^148^ that binds Ilp2 but not Ilp3^149^. Upregulation of *ImpL2* in tumours^114^ suggests it can selectively neutralise Ilp2, leaving Ilp3 available to promote tumour growth via niche signalling. Notably, unlike stem cell-specific inhibition of insulin signalling (e.g. *InR* or *Akt* knockdown), which fully abolishes proliferation differences, *Ilp3* loss does not completely normalise female tumour growth to male levels. This suggests partial redundancy with other Ilps, including Ilp5, but not Ilp2. We attempted to test this directly using *ilp2, ilp3, ilp5* triple mutants; however, these animals exhibit severely reduced viability, precluding analysis in our tumour context.

We show that female-biased Ilp3 signalling enhances gut stem cell activity and increases organ size, to support reproductive demands. This pathway is likely to be activated under environmental stress. For example, during reproductive diapause, triggered by cold or short photoperiods, ovarian development arrests and the midgut shrinks^150^. A recent study found this shrinkage to be female-specific and reversible, with *Ilp3* expression increasing during recovery^150^—implicating the Ilp3 pathway in adaptive gut resizing. Similarly, Ilp3 from visceral muscles drives gut remodelling during starvation^70^, a response that is likely sex-specific though only females were tested. Thus, Ilp3-mediated crosstalk between visceral muscles and stem cells not only optimises gut size for reproduction in females under baseline conditions but also enables sex-specific intestinal plasticity in response to nutrient deprivation, temperature, and photoperiod. Beyond the gut, Ilp3 is implicated in sex-biased physiology elsewhere. *Ilp3* expression in the brain is female-biased in response to sugar intake, linked to increased fat storage^151–153^, though functional validation is lacking. Under our dietary conditions, we observed no sex difference in brain *Ilp3* expression. Additionally, *Ilp3* knockout reduces female—but not male—pupal body size^154,155^, via unknown mechanisms.

In summary, we reveal a novel sex differentiation pathway that is present before tumour initiation and contributes to sex-specific organ size, supporting the broader principle that cellular sex drives normal physiological variation underlying cancer susceptibility, likely conserved throughout animal lineages.

## Methods

### Fly strains and media

#### Drivers

*esg-Gal4*^*NP7397*^*, UAS-GFP, Tub-Gal80*^*TS*^ chromosome^44^, *dsx*^*M*^*-LexA* (this study, see below for details), *VT063223-p65.AD* (BDSC: 75204, Flybase ID: FBti0190631), *R10G10-p65.AD* (BDSC: 70529, Flybase ID: FBst0070529), *CG10116-Gal4.DBD* (BDSC: 91399, Flybase ID: FBst0091399), *fru*^*P1*^*-Gal4* (BDSC: 66696, Flybase ID: FBst0066696), *fru*^*P1*^*-LexA* (BDSC: 66698, Flybase ID: FBst0066698), *yolk-Gal4* (BDSC: 58814, Flybase ID: FBst0058814), *GMR19B09-Gal4* (BDSC: 48840, Flybase ID: FBst0048840), *R15A01-p65.AD* (BDSC: 68837, Flybase ID: FBst0068837), *R71G01-Gal4.DBD* (BDSC: 69507, Flybase ID: FBst0069507), *Ilp3-Gal4* (gift from L. Erin O’Brien, generated in ref. ^117^, Flybase ID: FBtp0069556), *tubP-Gal80*^*TS*^ (BDSC: 7108, Flybase ID: FBst0007108), *GMR94E02-Gal4* (BDSC: 40684, Flybase ID: FBst0040684), *GMR46B08-Gal4* (BDSC: 47361, Flybase ID: FBst0047361), *GMR69H10-Gal4* (BDSC: 46626, Flybase ID: FBst0046626), *GMR18B03-Gal4* (BDSC: 48798, Flybase ID: FBst0048798), *esg-LacZ*^*k00606*^ (BDSC: 10359, Flybase ID: FBst0010359), *Ilp2-Gal4* (BDSC: 37516, Flybase ID: FBst0037516), *nSyb-GAL80* (BDSC: 92154, Flybase ID: FBst0092154), *Mef2-Gal4* (BDSC: 27390, Flybase ID: FBti0115746), *StenExSX-4, Tub-Gal80*^*TS*^*, LexAop-mCD8::GFP* chromosome (gift from Y. Kwon, generated in ref. ^115^, Flybase ID: FBti0185052), *GMR14D03-Gal4* (BDSC: 47463, Flybase ID: FBti0133237).

#### UAS transgenes

*UAS-tra*^*F*^ (BDSC: 4590, Flybase ID: FBst0004590), *UAS-InR* (BDSC: 8262, Flybase ID: FBst0008262), *UAS-Ilp3* (gift from H. Stocker, generated in ref. ^156^, Flybase ID: FBal0141675), *UAS-Ilp2* (BDSC: 80936, Flybase ID: FBst0080936), *UAS-PI3K92E*^*CAAX*^ (BDSC: 8294, Flybase ID: FBst0008294), *UAS-mCD8::GFP* (BDSC: *32185*, Flybase ID: *FBst0032185*)*, UAS-mCD8::GFP* (BDSC: 32186, Flybase ID: FBst0032186), *UAS-Stinger* (BDSC: 84277, Flybase ID: FBst0084277), *UAS-Stinger* (BDSC: 84278, Flybase ID: FBst0084278), *LexAop-grim-rpr* (gift from T. Lee, generated in ref. ^110^, Flybase ID: FBtp0165761), *LexAop-mCD8::GFP* (BDSC: 32203, Flybase ID: FBst0032203), *UAS-rpr* (BDSC: 5824, Flybase ID: FBst0005824), *UAS-brp.GFP* (BDSC: 36291, Flybase ID: FBst0036291), *LexAop-TrpA1* (Flybase ID: FBal0349259), *UAS-yki*^*3SA*^ (BDSC: 28817, Flybase ID: FBti0127382), *UAS-ovo*^*svb.ACT*^ (gift from C. Polesello, Flybase ID: FBtp0134164).

#### RNAi transgenes

*UAS-InR*^*RNAi*^ (VDRC: v992, Flybase ID: FBst0471628), *UAS-AkhR*^*RNAi*^ (BDSC: 51710, Flybase ID: FBst0051710), *UAS-Akt*^*RNAi#1*^ (BDSC: 33615, allele ID: FBst0033615), *UAS-Pten*^*RNAi*^ (BDSC: 33643, Flybase ID: FBst0033643), *UAS-gce*^*RNAi*^ (BDSC: 26323, Flybase ID: FBst0026323), *UAS-wit*^*RNAi*^ (BDSC: 41906, Flybase ID: FBst0041906), *UAS-put*^*RNAi*^ (BDSC: 35701, Flybase ID: FBst0035701), *UAS-babo*^*RNAi*^ (BDSC: 41585, Flybase ID: FBst0041585), *UAS-CCAPR*^*RNAi*^ (VDRC: v14767, Flybase ID: FBst0451587), *UAS-SPR*^*RNAi*^ (BDSC: 77389, Flybase ID: FBst0077389), *UAS-AstC-R2*^*RNAi*^ (BDSC: 36888, Flybase ID: FBst0036888), *UAS-AstC-R1*^*RNAi*^ (BDSC: 27506, Flybase ID: FBst0027506), *UAS-Met*^*RNAi*^ (BDSC: 61935, Flybase ID: FBst0061935), *UAS-Proc-R*^*RNAi*^ (BDSC: 29414, Flybase ID: FBst0029414), *UAS-TkR99D*^*RNAi*^ (BDSC: 27513, Flybase ID: FBst0027513), *UAS-CCHa1-R*^*RNAi*^ (BDSC: 27669, Flybase ID: FBst0027669), *UAS-NPFR*^*RNAi*^ (BDSC: 25939, Flybase ID: FBst0025939), *UAS-sNPFR*^*RNAi*^ (BDSC: 27507, Flybase ID: FBst0027507), *UAS-Dh31-R*^*RNAi*^ (BDSC: 25925, Flybase ID: FBst0025925), *UAS-Gyc76C*^*RNAi*^ (BDSC: 57315, Flybase ID: FBst0057315), *UAS-Crz*^*RNAi*^ (BDSC: 26017, Flybase ID: FBst0026017), *UAS-CapaR*^*RNAi*^ (BDSC: 27275, Flybase ID: FBst0027275), *UAS-PK2-R2*^*RNAi*^ (BDSC: 36827, Flybase ID: FBst0036827), *UAS-PK2-R1*^*RNAi*^ (BDSC: 29624, Flybase ID: FBst0029624), *UAS-MsR2*^*RNAi*^ (BDSC: 67869, Flybase ID: FBst0067869), *UAS-MsR1*^*RNAi*^ (BDSC: 27529, Flybase ID: FBst0027529), *UAS-CCHa2-R*^*RNAi*^ (BDSC: 25855, Flybase ID: FBst0025855), *UAS-AstA-R2*^*RNAi*^ (BDSC: 67864, Flybase ID: FBst0067864), *UAS-AstA-R1*^*RNAi*^ (BDSC: 27280, Flybase ID: FBst0027280), *UAS-TkR86C*^*RNAi*^ (BDSC: 31884, Flybase ID: FBst0031884), *UAS-FMRFaR*^*RNAi*^ (BDSC: 25858, Flybase ID: FBst0025858), *UAS-Pdfr*^*RNAi*^ (BDSC: 42508, Flybase ID: FBst0042508), *UAS-SIFaR*^*RNAi*^ (BDSC: 44068, Flybase ID: FBst0044068), *UAS-Lkr*^*RNAi*^ (BDSC: 65934, Flybase ID: FBst0065934), *UAS-sc*^*RNAi*^ (BDSC: 41594, Flybase ID: FBst0041594), *UAS-Ilp3*^*RNAi*^ (VDRC: v106512, Flybase ID: FBst0478336), *UAS-Notch*^*RNA i*^(VDRC: v27229, Flybase ID: FBst0456826), *UAS-Notch*^*RNAi*^ (VDRC: v100002, Flybase ID: FBst0471876), *LexAop-Notch*^*shRNA*^ (this study, see below for details), *UAS-Akt*^*RNAi#2*^ (VDRC: v2902, Flybase ID: FBti0083396), *UAS-Akt*^*RNAi#3*^ (NIG-Fly: 4006R-3, Flybase ID: FBti0246817), *UAS-tra2*^*RNAi*^ (BDSC: 28018, FlyBase ID: FBti0128004).

#### Mutants

*Ilp3*^*KO*^ (BDSC: 30882, Flybase ID: FBst0030882), *Ilp2*^*KO*^ (BDSC: 30881, Flybase ID: FBst0030881), *Ilp5*^*KO*^ (BDSC: 30884, Flybase ID: FBst0030884), *dsx*^1^ (BDSC: 1679, Flybase ID: FBst0001679), *dsx*^*Δ*^ (generated in ref. ^157^, Flybase ID: FBal0325111), *fru*^*V5*^ (gift from Y. Pan, generated in ref. ^113^, Flybase ID: FBal0366865), *fru*^*F*^ (BDSC: 66873, Flybase ID: FBst0066873), *Df(3 R)fru*^4–40^ (BDSC: 66692, Flybase ID: FBst0066692).

#### Feminising alleles

The following femising transgenic alleles were generated in this study (see below for details): *tra>tra*^*F*52^, *dsx>tra*^*F*^, *fru*^*P1*^*>tra*^*F*^, *Cg>tra*^*F*^*, CG10116>tra*^*F*^*, Ilp2>tra*^*F*^*, Ilp7>tra*^*F*^*, Dh44>tra*^*F*^*, CCAP>tra*^*F*^*, nSyb>tra*^*F*^, *Pdf>tra*^*F*^*, trh>tra*^*F*^*, Akh>tra*^*F*^*, R20C06>tra*^*F*^*, R76F12>tra*^*F*^*, R37F03>tra*^*F*^*, R22C05>tra*^*F*^, *R25F07>tra*^*F*^, *Glia>tra*^*F*^, *ARCEN>tra*^*F*^, and *Ilp3>tra*^*F*^.

Animals were reared on fly food containing (per liter): 10 g of agar, 82.5 g polenta, 34 g dry yeast and 3,75 g Moldex (per liter, diluted in ethanol). All experimental flies were kept in incubators at 25 °C, and on a 12 h light/dark cycle. Flies were transferred to fresh vials every 3 days, and fly density was kept to a maximum of 15 flies per vial.

### Generation of the *dsx*^*ΔM*^ CRISPR mutant and *dsx*^*M*^*-LexA* knock-in line

The *dsx*^*M*^*-LexA* line was generated in two steps. First, we created the *dsx*^*ΔM*^ allele, in which the male-specific coding exon of *dsx* was deleted. To generate this allele, two guide RNAs (gRNA1: aaaatggcgcacatcttggtcgg; gRNA2: atactgctacgtggcagccgtgg) targeting the flanking regions of the male-specific exon were cloned into the pCFD5 vector (Addgene plasmid #73914). A 0.938 kb left homology arm (flanking the gRNA1 cleavage site) was PCR-amplified from genomic DNA using Q5 High-Fidelity DNA Polymerase (NEB, M0491S), digested with EcoRI and SacII, and cloned into the pDsRed-attP vector (Addgene plasmid #51019). A 0.992 kb right homology arm (flanking the gRNA2 cleavage site) was similarly PCR-amplified, digested with SpeI and AscI, and cloned into the same vector already containing the left arm. The construct was sequence-verified and co-injected (BestGene) with the pCFD5-gRNA vector into *yw; nos-Cas9* embryos (FlyBase ID: FBti0156858). The resulting CRISPR-mediated deletion removed 1,025 bp of the *dsx* locus, including the male-specific exon, and replaced it with an attP landing site and a loxP-flanked 3xP3-DsRed marker. In a second step, to generate the *dsx*^*M*^*-LexA* knock-in allele, T2A-LexA sequences were cloned downstream of the previously deleted 1,025 bp *dsx* fragment into the RIV FRTnMCS1FRT-white vector (DGRC plasmid #1333). The construct was sequence-verified, and transgenic lines were generated via ΦC31 integrase-mediated transformation (BestGene), using the *dsx*^*MΔ*^ allele as the landing site.

### Generation of the *fru*^*P1*^*>tra*^*F*^ knock-in allele

To generate a knock-in of *tra*^*F*^ under the control of the *fru*^*P1*^ promoter, we performed recombination-mediated cassette exchange (RMCE) using the MiMIC insertion *Mi{y[+mDint2]=MIC}fru[MI05459]* (BDSC: 42086; FlyBase ID: FBti0149046). Embryos carrying this insertion were injected with a midiprep preparation of the donor plasmid *pBS-KS-attB2-SA(1)-T2A-tra*^*F*^*-Hsp70*, along with a ΦC31 integrase-expressing plasmid. The donor plasmid was derived from Addgene plasmid #62952 by replacing the *Gal80* coding sequence with the *traF* coding region (687 nt; FlyBase ID: FBtr0075364). A *3xP3-DsRed* marker was also added to facilitate the identification of transformants. The orientation of the inserted *tra*^*F*^ exon was confirmed by PCR amplification.

### Generation of the *dsx>tra*^*F*^ knock-in allele

To generate a knock-in of *tra*^*F*^ under the control of the *dsx* regulatory sequences, we performed a similar RMCE strategy using the MiMIC insertion *Mi{y[+mDint2]=MIC}dsx[MI03050]* (BDSC: 36182, FlyBase ID: FBti0143242). Embryos carrying this insertion were injected with a midiprep preparation of the donor plasmid *pBS-KS-attB2-SA(1)-T2A-tra*^*F*^*-Hsp70*, along with a ΦC31 integrase-expressing plasmid.

### Generation of the eighteen tissue-specific enhancers *tra*^*F*^ lines

To express Tra^F^ in specific organs or cell types independently of the Gal4-UAS system, we generated transgenic lines in which tissue-specific enhancers were cloned upstream of the *Drosophila* synthetic core promoter and the *tra*^*F*^ coding sequence (687-nt; FlyBase ID: FBtr0075364). Enhancer sequences were synthesised (GenScript) and inserted into the RIV FRTnMCS1FRT-white vector (DGRC Plasmid #1333), which includes one CTCF and one gypsy insulator upstream of the enhancer. The following regulatory regions were cloned: *Cg>tra*^*F*^: a 2770-nt intergenic region between *Col4a1* and *vkg*^81^, *CG10116>tra*^*F*^: a 951-nt region upstream of *CG10116*^58^, *Ilp3>tra*^*F*^: a 1,350-nt region between *Ilp4* and *Ilp3*^117^, *Ilp2>tra*^*F*^: the regulatory region located between *Ilp1* and *Ilp2, Ilp7>tra*^*F*^: a 1,600-nt fragment upstream of the *Ilp7* start codon, *Pdf>tra*^*F*^: an 813-nt region between *CG14237* and the *Pdf* start codon, *Akh>tra*^*F*^: a 188-nt fragment between *Ras24B* and the *Akh* start codon, *Dh44>tra*^*F*^: an 800-nt region upstream of the *Dh44* transcription start site, and *CCAP>tra*^*F*^: the region between *CG6972* and the *CCAP* start codon. Additional *tra*^*F*^ lines were generated using characterised enhancer fragments: *R20C06>tra*^*F*^: GMR20C06 (FlyBase ID: FBsf0000162528), a 2,542-nt intronic fragment from *nAChRα5* (35485968), *nSyb>tra*^*F*^: GMR57C10 (FlyBase ID: FBsf0000165807), an 824-nt fragment from *nSyb*, including its putative endogenous promoter, *trh>tra*^*F*^: GMR14D03 (FlyBase ID: FBsf0000162001), a 2220-nt intronic fragment from *trh*^80^, *Glia>tra*^*F*^: GMR56F03 (FlyBase ID: FBsf0000165743), a 2644-nt fragment from *rumpel* including its promoter, driving expression in ensheathing glia^82^, *ARCEN>tra*^*F*^: GMR49E06 (FlyBase ID: FBsf0000165107), a 3,647-nt fragment from *trabuco* including its promoter, driving expression in cholinergic gut-innervating neurons^89^, *R76F12>tra*^*F*^: GMR76F12 (FlyBase ID: FBsf0000167105), a 530-nt intronic fragment from *amon* driving expression in neurons projecting to the posterior midgut^90^, *R37F03>tra*^*F*^: GMR37F03 (FlyBase ID: FBsf0000164074), a 1,793-nt intronic fragment from *Scp2*, also driving expression in posterior midgut-projecting neurons^90^, *R22C05>tra*^*F*^: GMR22C05 (FlyBase ID: FBsf0000162709), a 2985-nt intronic fragment from *fru*, driving expression in posterior midgut-projecting neurons^90^, and *R25F07>tra*^*F*^: GMR25F07 (FlyBase ID: FBsf0000163024), a 1868-nt intronic fragment from *Fur1*, driving expression in neurons projecting to the posterior midgut^90^. All constructs were sequence-verified and integrated into the *Drosophila* genome via ΦC31 integrase-mediated transformation (BestGene). Full plasmid sequences and constructs are available upon request. The *Cg>tra*^*F*^*, CG10116>tra*^*F*^*, Ilp2>tra*^*F*^*, Ilp7>tra*^*F*^*, Dh44>tra*^*F*^*, CCAP>tra*^*F*^*, nSyb>tra*^*F*^, and *trh>tra*^*F*^ constructs were inserted into a recently generated amorphic *tra* allele^44^ in which the endogenous *tra* locus is replaced by an attP site (BDSC: 67412; FlyBase ID: FBti0186559). The *Pdf>tra*^*F*^*, Akh>tra*^*F*^*, R20C06>tra*^*F*^*, R76F12>tra*^*F*^*, R37F03>tra*^*F*^*, R22C05>tra*^*F*^, and *R25F07>tra*^*F*^ constructs were integrated into the VK00005 attP site line (BDSC: 9725; FlyBase ID: FBti0076428). The *Glia>tra*^*F*^ and *ARCEN>*^*traF*^ transgenes were introduced into the attP2 site line (BDSC: 8622; FlyBase ID: FBti0040535), while *Ilp3>tra*^*F*^ was inserted into the VK00014 site line (BDSC: 9733; FlyBase ID: FBti0076436).

### Generation of the *LexAop-Notch*^*shRNA*^ line

To generate this line, a *shRNA* (TRiP.HMS00001, FlyBase ID: FBti0140084, sequence: GCGGCGGTTAACAATACCGAA) targeting *Notch* was cloned by gene synthesis (Genscript) into the pWALEXA20 vector (DGRC Stock Number#1584) between the XbaI and NdeI restrictions sites. The construct was sequence-verified and transgenic lines were established through ΦC-31 integrase mediated transformation (Bestgene), using the attP-9A-VK00027 (BDSC: 9744, FlyBase ID: FBti0076447) attP site line.

### RNA-seq

The RNA-seq transcriptional data of adult midguts obtained from males, females, and *tra* mutant females used for Fig. 2b and Supplementary Fig. 3a is available from GEO under accession numbers: GSE74772, and GSE74774.

### Reverse transcription and quantitative PCR

Total RNA was extracted from 20 dissected midguts (excluding the crop) using the RNeasy Mini Kit (Qiagen), or from 20 heads using the RNeasy Lipid Tissue Mini Kit (Qiagen). Genomic DNA was removed, and complementary DNA (cDNA) was synthesized from 1000 ng of total RNA using the iScript gDNA Clear cDNA Synthesis Kit (Bio-Rad). Quantitative PCR (qPCR) was performed using iTaq Universal SYBR Green Supermix (Bio-Rad, 172-5124) and gene-specific primers in 384-well plates. cDNA input was 40 ng for assays measuring *Ilp* expression in gut and head, and 50 ng for *Ilp3* expression in flies with feminised or ablated *fru*^*P1*^ neurons. Relative gene expression was normalized to *rp49* as a reference gene. For visualisation, the mean expression level in control males was arbitrarily set to 100%, and expression levels in other sexes and genotypes are presented as a percentage of this baseline. The following qPCR primer pairs were used: *rp49* (5′-CTTCATCCGCCACCAGTC-3′, 5′-CGACGCACTCTGTTGTCG-3′), *Ilp2* (5’-ATGGTGTGCGAGTATAATCC-3’, 5’-TCGGCACCGGGCATG-3’), *Ilp3* (5’-AGAGAACTTTGGACCCCGTGAA-3’, 5’-TGAACCGAACTATCACTCAACAGTCT-3’), *Ilp5* (5’-TGCCTGTCCCAATGGATTCAA-3’, 5’-GCCAAGTGGTCCTCATAATCG-3’), *fru*^*F*^ (5′-GTCCATCGCTCCTTGGTCAGTGT-3′, 5′-CCTCAAAAGCCCGGCAACCT-3′), *fru*^*M*^ (5′-GTACGGACTGCAGGCTGACAGGA-3′, 5′-GCCCGCATCCCCTAGGTACAACA-3′), *dsx*^*F*^ (5′-ACATTGCCGCGTTGTGTTGCG-3′, 5′-GCGAATCGAAGAGGGCCAA-3′), *dsx*^*M*^ (5′-ACCAGGGCCATCGGGGTGTA-3′, 5′-GGCTTCCCGGCGAATCGAAG-3′), *Yp1* (5′-CAACTCCGTCAACCAGGCATT-3′, 5′-GACAGGTGGTAGACTTGCTGC-3′), *tra*^*F*^ (5′-AACCCAGCATCGAGATTCCC-3′, 5′-ACCTCGTCTGCAAAGTACGG-3′).

### Immunofluorescence on adult tissues

Tissues were dissected in PBS, fixed in 4% formaldehyde (Polysciences) in PBS for 30 minutes at room temperature (RT), and then washed several times in PBS containing 0.3% Triton X-100 (PBT). Samples were blocked in PBT supplemented with 10% foetal bovine serum for 1 h at RT. Dissected tissues were incubated with primary antibodies for two nights at 4 °C. Following several washes in PBT, tissues were incubated with secondary antibodies for 3 h at RT. After a 20-min incubation with DAPI, samples were mounted in Vectashield (Vector Laboratories). Fluorescence images were acquired using a Leica SP5 DS confocal microscope. The following primary antibodies and reagents were used: Rabbit anti-Phospho-Histone H3 (Ser10) (1:500, Cell Signalling #9701), Chicken anti-GFP (1:10,000, Abcam ab13970), Mouse anti-Prospero (1:10, DSHB MR1A), Chicken anti-βGal (1:1,000, GeneTex GTX77365), Rat anti-FruM (Clone #1C1, 1:10; gift from K. Sato), Rhodamine Phalloidin (1:1,000, ThermoFisher Scientific), Rat anti-Ilp2 (1:400; gift from P. Léopold) and Rabbit anti-V5-Tag (D3H8Q) (1:100, Ozyme).

### Generation of genetically induced midgut tumours

Midgut tumours were induced using flies carrying the *esg-Gal4*^*NP7397*^*, UAS-GFP, Tub-Gal80*^*TS*^ chromosome in combination with a *UAS-Notch*^*RNAi*^ transgene. Flies were raised at 18 °C, aged for 3–5 days and shifted to 29 °C at the adult stage for 14 days to activate transgene expression. Tumour incidence was defined as the number of midguts containing a cluster of GFP-positive cells with at least one phospho-Histone H3 (pH3)-positive mitotic cell, per experimental replicate. Mitotic activity per midgut was quantified by counting the total number of pH3-positive cells. For consistency, counts were capped at 150 pH3-positive cells per gut, as tumours exceeding this threshold had already invaded the entire midgut.

For damage-induced regeneration assays, flies were transferred to empty vials containing a piece of 3.75 cm × 2.5 cm filter paper as a feeding substrate. The paper was soaked with 500 μL of either 5% sucrose solution (control) or 5% sucrose supplemented with 3% dextran sulphate sodium (DSS). Flies were transferred daily to fresh vials with newly soaked paper for three consecutive days prior to dissection.

### Fertility tests

For fertility assays, males and females were collected upon eclosion and aged for 3–5 days. In female fertility experiments, five experimental females were mated with five Canton-S males per vial for five days. In male fertility experiments, a single experimental male was mated with five Canton-S females per vial for five days. After this period, adult flies were removed, and progeny were counted to assess fertility.

### Statistics and data presentation

All statistical analyses were performed using the software GraphPad Prism 10.1.1.

### Reporting summary

Further information on research design is available in the Nature Portfolio Reporting Summary linked to this article.

## Supplementary information

Supplementary Information

Peer Review file

Reporting Summary

## Source data

Source Data

## Data Availability

All data is available in the main text or the supplementary materials. Materials generated for the study are available from the corresponding authors on request. Source data are provided with this paper.
