# Supplementary material for: Sex of central neurons controls intestinal niche signalling to drive sex differences in stem cell activity and cancer susceptibility
Source: Nat Commun. 2026 Aug 22;17:10043. doi: 10.1038/s41467-026-76998-z (PMC13594308; doi:10.1038/s41467-026-76998-z)
Supplement: Supplementary file 1 [file 41467_2026_76998_MOESM1_ESM.pdf]

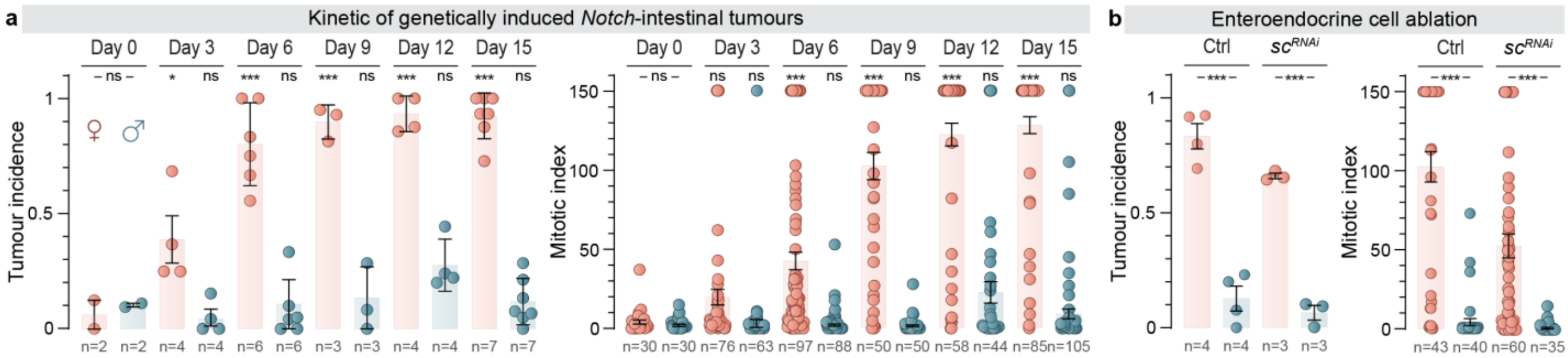

Supplementary figure 1. Sex-biased intestinal tumorigenesis is independent of enteroendocrine cell presence.
a, Quantification of tumour incidence and neoplastic growth (pH3⁺ cell number) in adult midguts of females and males following *Notch* silencing in progenitor cells during different time windows. *p = 0.0277, ***p < 0.0001 (one-way ANOVA). **b**, Quantification of tumour incidence and neoplastic growth (pH3⁺ cell number) in adult midguts upon *Notch* silencing in progenitors, with or without *scute (sc)* knock-down. ***p < 0.0001 (one-way ANOVA). In all panels, females are shown in red and males in blue. For tumour incidence, n=number of experiments; for mitotic index, n=number of midguts. Data from ≥3 independent experiments; bars represent mean ± SEM.

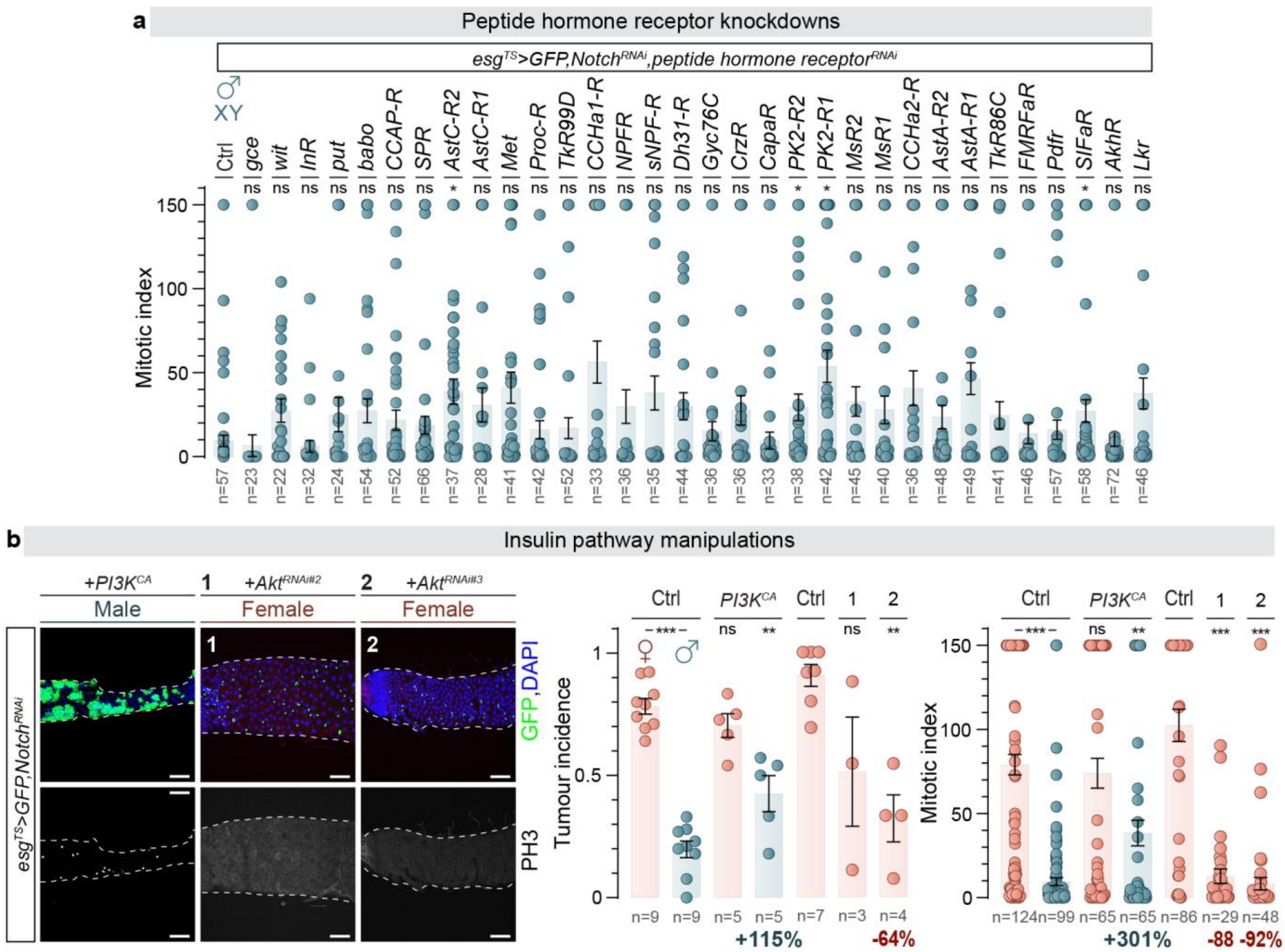

Supplementary figure 2. Sexual identity of non- intestinal cells drives insulin-dependent tumour expansion.
a, Neoplastic growth (pH3⁺ cell number) in male midguts upon progenitor-specific *Notch* silencing combined with individual peptide hormone receptor knockdowns. *p < 0.0409 (one-way ANOVA). **b**, Tumour incidence and neoplastic growth in adult midguts of both sexes following expression of a constitutively active form of *Phosphatidylinositol 3-kinase 92E* (*Pi3K^{CA}*) in progenitor cells (which acts downstream of InR and upstream of Akt), or a *RNAi* targeting *Akt* (*Akt$^{RNAi\#2}$* (condition 1) or *Akt$^{RNAi\#3}$* (condition 2)). Confocal images show tumour coverage in the R4 region (DAPI: DNA, blue; GFP: progenitors, green; pH3: dividing cells, grey). **p < 0.0047, ***p < 0.0001 (one-way ANOVA). Scale bars: 50µm.

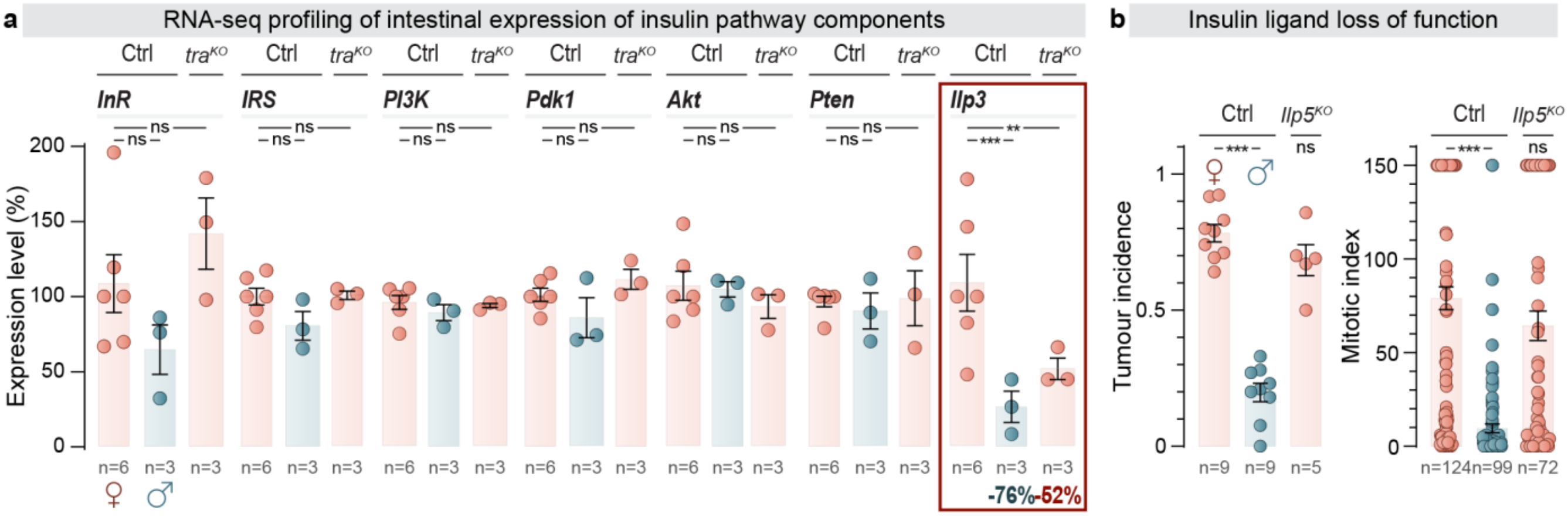

Supplementary figure 3. Ilp3 is a female-enriched, niche-derived driver of gut tumour growth.
a, Normalised expression levels of core components of the Insulin signalling pathway in the adult intestine of control females, males, and *tra*^{KO} females, based on transcriptional datasets from[44] (GSE74772, GSE74774), including the insulin receptor *InR*, its substrate *IRS* linking InR to downstream signalling, the kinases *PI3K*, *Pdk1*, and *Akt*, and the negative regulator *Pten*. **b**, Tumour incidence and neoplastic growth (pH3^{+} cell number) in midguts with progenitor-specific *Notch* silencing combined with ubiquitous *Ilp5* knock-out. ***p < 0.0001 (one-way ANOVA).

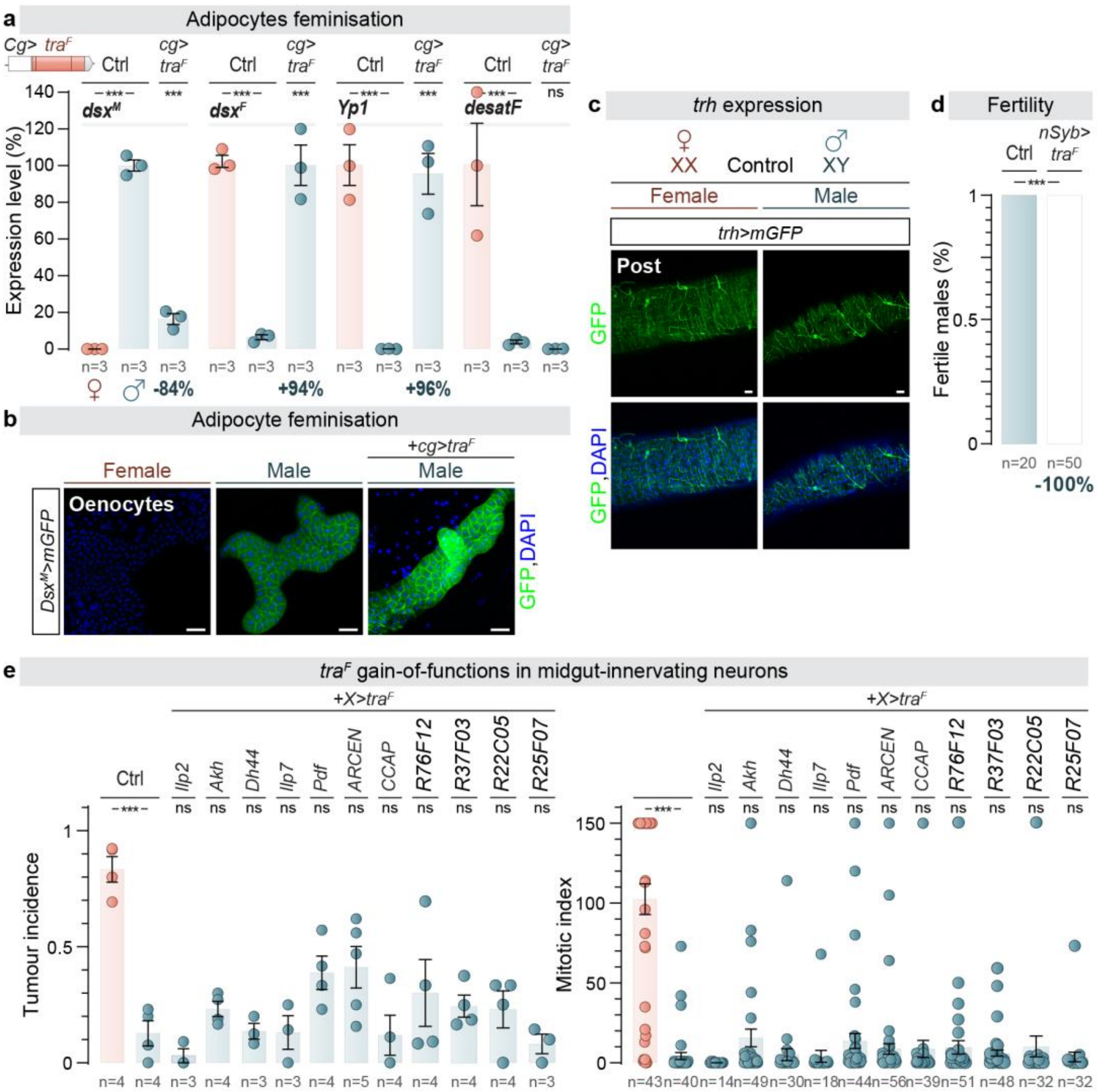

Supplementary figure 4. Neuronal sex dictates sexual dimorphism in gut tumours.
a, RT-qPCR expression data of sex-specific *dsx* isoforms (*dsx*^{M} and *dsx*^{F}), the Dsx^{F} target genes, *Yolk protein 1* (*Yp1*) and *desatF* in control and *cg>tra*^{F} males. ***p < 0.0003 (one-sided t-test). **b**, Expression of *dsx*$^{M\text{-}LexA}$ reporter in adult oenocytes of control and males expressing *tra*^{F} in adipocytes (*Cg>tra*^{F}) (DAPI: DNA, blue; reporter-positive cells: mGFP, green). **c**, Expression of the tracheal-specific enhancer of *trh* (*GRM14D03 - trh>*) in adult intestines of control males and females (DAPI: DNA, blue; *trh>mGFP*, green). **d**, Fertility assay showing the number of fertile individuals in controls and males expressing *tra*^{F} in neurons (*nSyb>tra*^{F}). ***p < 0.0001 (one-way ANOVA). **e**, Tumour incidence and neoplastic growth in adult intestines following progenitors-specific *Notch* silencing combined with *tra*^{F} expression in selected gut-innervating neurons (*X>tra*^{F}). ***p < 0.0001 (one-way ANOVA).

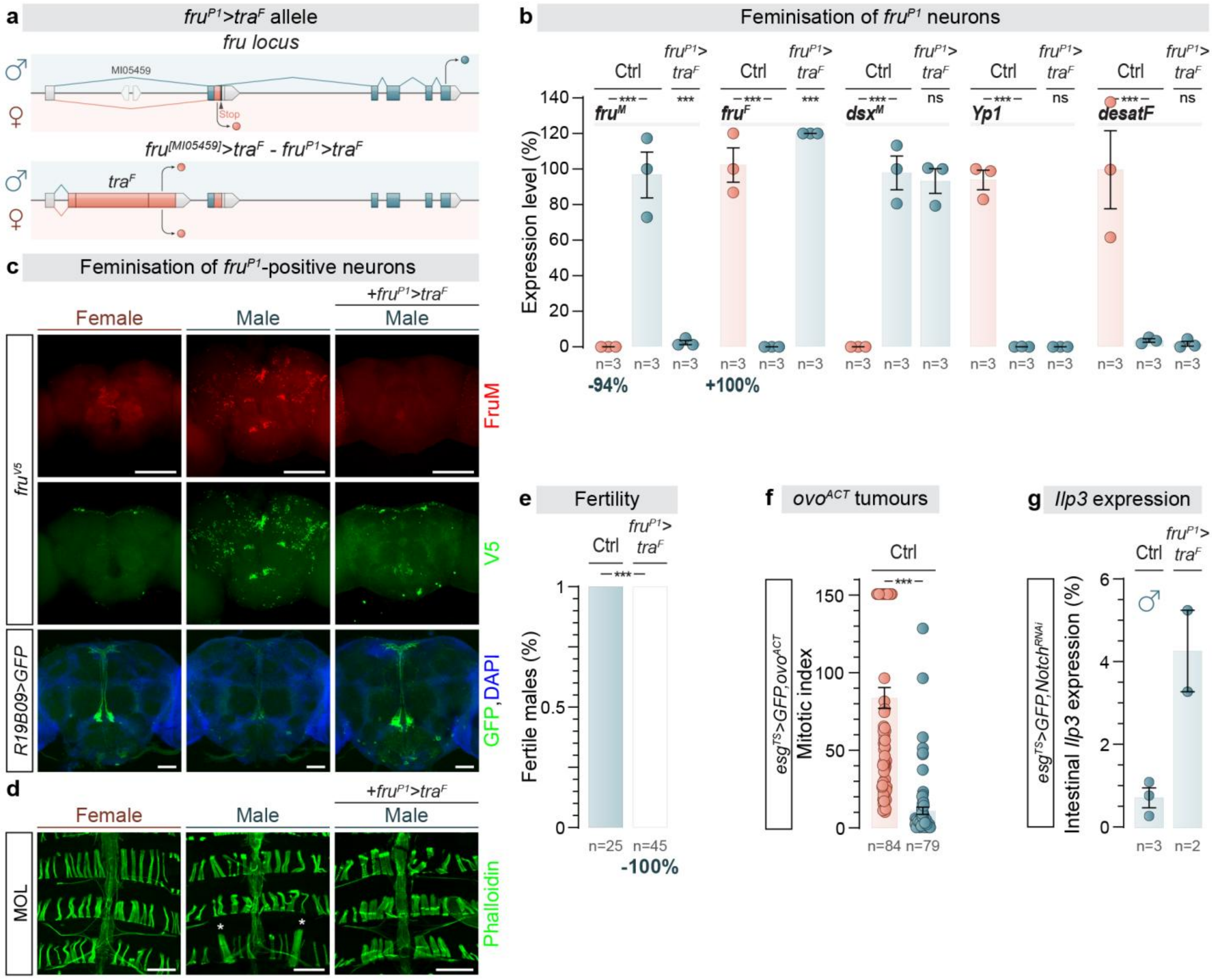

Supplementary figure 5. Sexual identity of *fruitless*^{P1} neurons governs intestinal tumour sex bias.

a, Schematic of the *fruitless* (*fru*) genomic locus and the newly generated *fru*^{P1}*>tra*^{F} allele produced via MiMIC cassette swapping in the *fru[MI05459]* background. **b**, RT-qPCR analysis of sex-specific *fru/dsx* isoforms (*fru*^{M}, *fru*^{F}, *and dsx*^{M}), the Dsx^{F} target genes, *Yolk protein 1* (*Yp1*) and *desatF* in control and *fru*^{P1}*>tra*^{F} males. ***p < 0.0001 (one-sided t-test). **c**, Expression of the male-specific Fru^{M} proteins in adult brains of control flies and *fru*^{P1}*>tra*^{F} males visualised using either anti-Fru^{M} antibody or anti-V5 antibody in a V5-tagged knock-in line (Fru^{M}, red; V5, green). Visualisation of female-specific *R19B09*-positive neurons in brains of control and *fru*^{P1}*>tra*^{F} males (DAPI: DNA, blue; *R19B09*>GFP, green). **d**, Dorsal musculatures of control and *fru*^{P1}*>tra*^{F} males. The male-specific muscles of Lawrence (MOL) are indicated by asterisks (Phalloidin staining for actin, green). **e**, Fertility assay showing the number of fertile individuals in controls and males expressing *tra*^{F} in *fru*^{P1}-neurons (*fru*^{P1}*>tra*^{F}). ***p < 0.0001 (one-way ANOVA). **f**, Neoplastic growth following progenitor-specific overexpression of a constitutive active from of the transcription factor *ovo/shavenbaby (ovo*^{ACT}*).* ***p < 0.0001, *p=0.01 (one-way ANOVA). **g**, RT-qPCR analysis of *Ilp3* expression in adult midguts after progenitor-specific *Notch* silencing in controls, and *fru*^{P1}*>tra*^{F} males. Scale bars: 50 µm.

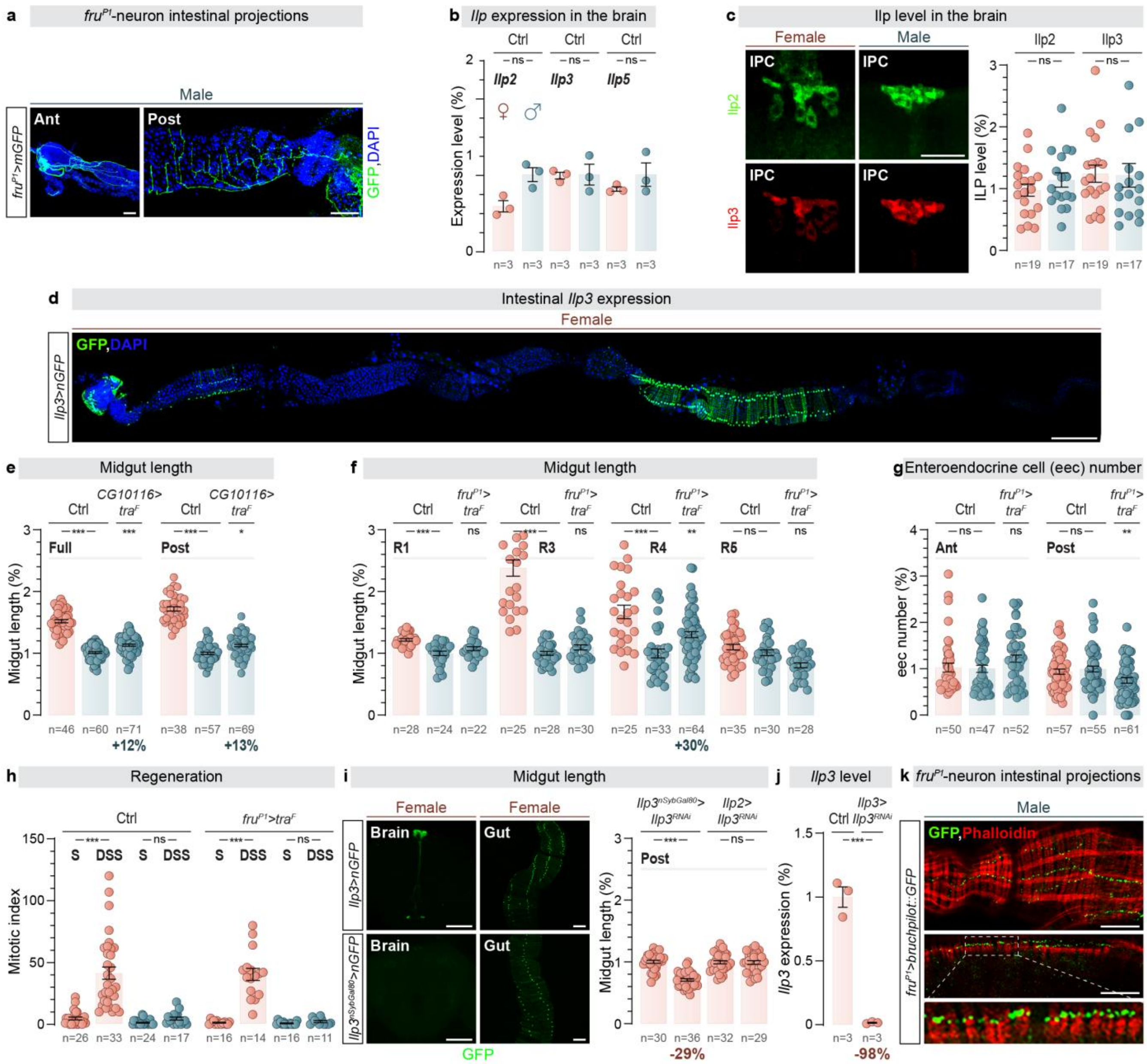

Supplementary figure 6. Sexual identity of *fru^{P1}* neurons orchestrates Ilp3-dependent regulation of gut size and fecundity.

a, Visualisation of enteric projections of *fru^{P1}* neurons in control males (DAPI: DNA, blue; *fru^{P1}*>GFP, green). **b**, RT-qPCR analysis of *Ilp2*, *Ilp3*, and *Ilp5* expression in adult brains of males, and females. **c**, Protein expression level of Ilp2, and Ilp3 in adult brains of control flies, visualised using anti-Ilp2 or anti-Ilp3 antibodies. **d**, Visualisation of *Ilp3* expression in the anterior R1 and posterior R4 midgut regions using the *Ilp3^{Gal4}* reporter line (DAPI: DNA, blue; *Ilp3*>GFP, green). **e**, Quantification of total and posterior midgut length in control and *CG10116>tra^F* males. *p = 0.0141, ***p < 0.0004 (one-way ANOVA). **f**, Quantification of region-specific midgut length in controls and *fru^{P1}>tra^F* males. **p = 0.0032, ***p < 0.0001 (one-way ANOVA). **g**, Quantification of enteroendocrine cell number (Pros-positive cells) in the anterior and posterior regions of control and *fru^{P1}>tra^F* males. **p = 0.0086 (one-way ANOVA). **h**, Proliferative response of intestinal progenitors (pH3$^+$ cell count) following exposure to control (sucrose, S) or damage-inducing (DSS) diets in controls and *fru^{P1}>tra^F* males. ***p < 0.0001 (one-way ANOVA). **i**, Midgut length quantification in flies with *Ilp3* silencing in the visceral muscles only (*Ilp3^{nSybGal80}*), and the insulin-producing cells (IPCs) only (*Ilp2>*). Representative confocal images show *Ilp3*>nGFP and *Ilp3^{nSybGal80}>nGFP* reporter expressions in the visceral musculature and the IPCs (Ilp3>GFP, green). ***p < 0.0001 (one-way ANOVA). **j**, RT-qPCR analysis of *Ilp3* expression in whole bodies of control and *Ilp3>ilp3^{RNAi}* females. **k**, Visualisation of the neuro-muscular junctions formed by the *fru^{P1}* neurons in control males using the pre-synaptic marker: Bruchpilot (Phalloidin: F-Actin, red; *fru^{P1}*>bruchpilot::GFP, green). Scale bars: a, 50µm; c, 10 µm; d, 200 µm; i, 10µm and i, 50 µm; k, 50 µm.

Table S1. Fly stocks used in this study

Figure 1.

Figure 1a.

w*;esg-Gal4^{NP7397},UAS-GFP,Tub-Gal80^{TS}/+;UAS-Notch^{RNAi} (VDRC: v27229)/+
w*;esg-Gal4^{NP7397},UAS-GFP,Tub-Gal80^{TS}/+;UAS-Notch^{RNAi} (VDRC: v27229)/tra>traF

Figure 1b.

w*;esg-Gal4^{NP7397},UAS-GFP,Tub-Gal80^{TS}/+;UAS-Notch^{RNAi} (VDRC: v27229)/+
w*;esg-Gal4^{NP7397},UAS-GFP,Tub-Gal80^{TS}/UAS-tra^{F} (BDSC: 4590);UAS-Notch^{RNAi} (VDRC: v27229)/+
w*;esg-Gal4^{NP7397},UAS-GFP,Tub-Gal80^{TS}/+;UAS-Notch^{RNAi} (VDRC: v27229)/R20C06>traF
w*;esg-Gal4^{NP7397},UAS-GFP,Tub-Gal80^{TS}/+;UAS-Notch^{RNAi} (VDRC: v27229)/CG10116>traF

Figure 1c.

w*;;dsx^{M}-LexA,LexAop-mCD8::GFP (BDSC: 32203)/+
w*;;dsx^{M}-LexA,LexAop-mCD8::GFP (BDSC: 32203)/CG10116>traF
w* ;VT063223-p65.AD (BDSC: 75204)/CG10116-Gal4.DBD (BDSC: 91399);UAS-Stinger (BDSC: 84278)/+
w* ;VT063223-p65.AD (BDSC: 75204)/CG10116-Gal4.DBD (BDSC: 91399);UAS-Stinger (BDSC: 84278)/CG10116>traF
w* ; R10G10-p65.AD (BDSC: 70529)/CG10116-Gal4.DBD (BDSC: 91399);UAS-Stinger (BDSC: 84278)/+
w* ; R10G10-p65.AD (BDSC: 70529)/CG10116-Gal4.DBD (BDSC: 91399);UAS-Stinger (BDSC: 84278)/CG10116>traF

Figure 2.

Figure 2a.

w*;esg-Gal4^{NP7397},UAS-GFP,Tub-Gal80^{TS}/+;UAS-Notch^{RNAi} (VDRC: v27229)/+
w*;esg-Gal4^{NP7397},UAS-GFP,Tub-Gal80^{TS}/+;UAS-Notch^{RNAi} (VDRC: v27229)/UAS-gce^{RNAi} (BDSC: 26323)
w*;esg-Gal4^{NP7397},UAS-GFP,Tub-Gal80^{TS}/+;UAS-Notch^{RNAi} (VDRC: v27229)/UAS-wit^{RNAi} (BDSC: 41906)
w*;esg-Gal4^{NP7397},UAS-GFP,Tub-Gal80^{TS}/+;UAS-Notch^{RNAi} (VDRC: v27229)/UAS-InR^{RNAi} (VDRC: v992)
w*;esg-Gal4^{NP7397},UAS-GFP,Tub-Gal80^{TS}/UAS-put^{RNAi} (BDSC: 35701);UAS-Notch^{RNAi} (VDRC: v27229)/+
w*;esg-Gal4^{NP7397},UAS-GFP,Tub-Gal80^{TS}/+;UAS-Notch^{RNAi} (VDRC: v27229)/UAS-babo^{RNAi} (BDSC: 41585)
w*;esg-Gal4^{NP7397},UAS-GFP,Tub-Gal80^{TS}/+;UAS-Notch^{RNAi} (VDRC: v27229)/UAS-CCAPR^{RNAi} (VDRC: v14767)
w*;esg-Gal4^{NP7397},UAS-GFP,Tub-Gal80^{TS}/UAS-SPR^{RNAi} (BDSC: 77389);UAS-Notch^{RNAi} (VDRC: v27229)/+
w*;esg-Gal4^{NP7397},UAS-GFP,Tub-Gal80^{TS}/+;UAS-Notch^{RNAi} (VDRC: v27229)/UAS-AstC-R2^{RNAi} (BDSC: 36888)
w*;esg-Gal4^{NP7397},UAS-GFP,Tub-Gal80^{TS}/+;UAS-Notch^{RNAi} (VDRC: v27229)/UAS-AstC-R1^{RNAi} (BDSC: 27506)
w*;esg-Gal4^{NP7397},UAS-GFP,Tub-Gal80^{TS}/+;UAS-Notch^{RNAi} (VDRC: v27229)/UAS-Met^{RNAi} (BDSC: 61935)
w*;esg-Gal4^{NP7397},UAS-GFP,Tub-Gal80^{TS}/+;UAS-Notch^{RNAi} (VDRC: v27229)/UAS-Proc-R^{RNAi} (BDSC: 29414)
w*;esg-Gal4^{NP7397},UAS-GFP,Tub-Gal80^{TS}/+;UAS-Notch^{RNAi} (VDRC: v27229)/UAS-TkR99D^{RNAi} (BDSC: 27513)
w*;esg-Gal4^{NP7397},UAS-GFP,Tub-Gal80^{TS}/+;UAS-Notch^{RNAi} (VDRC: v27229)/UAS-CCHa1-R^{RNAi} (BDSC: 27669)
w*;esg-Gal4^{NP7397},UAS-GFP,Tub-Gal80^{TS}/+;UAS-Notch^{RNAi} (VDRC: v27229/UAS-NPFR^{RNAi} (BDSC: 25939)
w*;esg-Gal4^{NP7397},UAS-GFP,Tub-Gal80^{TS}/+;UAS-Notch^{RNAi} (VDRC: v27229)/UAS-sNPFR^{RNAi} (BDSC: 27507)
w*;esg-Gal4^{NP7397},UAS-GFP,Tub-Gal80^{TS}/+;UAS-Notch^{RNAi} (VDRC: v27229)/UAS-Dh31-R^{RNAi} (BDSC: 25925)
w*;esg-Gal4^{NP7397},UAS-GFP,Tub-Gal80^{TS}/+;UAS-Notch^{RNAi} (VDRC: v27229)/UAS-Gyc76C^{RNAi} (BDSC: 57315)
w*;esg-Gal4^{NP7397},UAS-GFP,Tub-Gal80^{TS}/+;UAS-Notch^{RNAi} (VDRC: v27229)/UAS-CrzR^{RNAi} (BDSC: 26017)
w*;esg-Gal4^{NP7397},UAS-GFP,Tub-Gal80^{TS}/+;UAS-Notch^{RNAi} (VDRC: v27229)/UAS-CapaR^{RNAi} (BDSC: 27275)
w*;esg-Gal4^{NP7397},UAS-GFP,Tub-Gal80^{TS}/+;UAS-Notch^{RNAi} (VDRC: v27229)/UAS-PK2-R2^{RNAi} (BDSC: 36827)
w*;esg-Gal4^{NP7397},UAS-GFP,Tub-Gal80^{TS}/+;UAS-Notch^{RNAi} (VDRC: v27229)/UAS-PK2-R1^{RNAi} (BDSC: 29624)
w*;esg-Gal4^{NP7397},UAS-GFP,Tub-Gal80^{TS}/UAS-MsR2^{RNAi} (BDSC: 67869);UAS-Notch^{RNAi} (VDRC: v27229)/+
w*;esg-Gal4^{NP7397},UAS-GFP,Tub-Gal80^{TS}/+;UAS-Notch^{RNAi} (VDRC: v27229)/UAS-MsR1^{RNAi} (BDSC: 27529)
w*;esg-Gal4^{NP7397},UAS-GFP,Tub-Gal80^{TS}/+;UAS-Notch^{RNAi} (VDRC: v27229)/UAS-CCHa2-R^{RNAi} (BDSC: 25855)
w*;esg-Gal4^{NP7397},UAS-GFP,Tub-Gal80^{TS}/UAS-AstA-R2^{RNAi} (BDSC: 67864);UAS-Notch^{RNAi} (VDRC: v27229)/+
w*;esg-Gal4^{NP7397},UAS-GFP,Tub-Gal80^{TS}/+;UAS-Notch^{RNAi} (VDRC: v27229)/UAS-AstA-R1^{RNAi} (BDSC: 27280)
w*;esg-Gal4^{NP7397},UAS-GFP,Tub-Gal80^{TS}/+;UAS-Notch^{RNAi} (VDRC: v27229)/UAS-TkR86C^{RNAi} (BDSC: 31884)
w*;esg-Gal4^{NP7397},UAS-GFP,Tub-Gal80^{TS}/+;UAS-Notch^{RNAi} (VDRC: v27229)/UAS-FMRFaR^{RNAi} (BDSC: 25858)
w*;esg-Gal4^{NP7397},UAS-GFP,Tub-Gal80^{TS}/UAS-Pdfr^{RNAi} (BDSC: 42508);UAS-Notch^{RNAi} (VDRC: v27229)/+
w*;esg-Gal4^{NP7397},UAS-GFP,Tub-Gal80^{TS}/UAS-SIFaR^{RNAi} (BDSC: 44068);UAS-Notch^{RNAi} (VDRC: v27229)/+
w*;esg-Gal4^{NP7397},UAS-GFP,Tub-Gal80^{TS}/UAS-AkhR^{RNAi} (BDSC: 51710) ;UAS-Notch^{RNAi} (VDRC: v27229)/+
w*;esg-Gal4^{NP7397},UAS-GFP,Tub-Gal80^{TS}/+;UAS-Notch^{RNAi} (VDRC: v27229)/UAS-Lkr^{RNAi} (BDSC: 65934)

Figure 2b.

w*;esgGal4^{NP7397},UAS-GFP,Tub-Gal80^{TS}/+;Df(3L)$^{st-j7}$/+
w*;esgGal4^{NP7397},UAS-GFP,Tub-Gal80^{TS}/+;Df(3L)$^{st-j7}$/tra^{KO}

Figure 2c.
w*;esg-Gal4^{NP7397},UAS-GFP,Tub-Gal80^{TS}/+;UAS-Notch^{RNAi} (VDRC: v27229)/+
w*;esg-Gal4^{NP7397},UAS-GFP,Tub-Gal80^{TS}/+;UAS-Notch^{RNAi} (VDRC: v27229)/UAS-InR^{RNAi} (VDRC: v992)
w*;esg-Gal4^{NP7397},UAS-GFP,Tub-Gal80^{TS}/+;UAS-Notch^{RNAi} (VDRC: v27229)/UAS-Akt^{RNAi} (BDSC: 33615)
w*;esg-Gal4^{NP7397},UAS-GFP,Tub-Gal80^{TS}/UAS-InR (BDSC: 8262);UAS-Notch^{RNAi} (VDRC: v27229)/+
w*;esg-Gal4^{NP7397},UAS-GFP,Tub-Gal80^{TS}/+;UAS-Notch^{RNAi} (VDRC: v27229)/UAS-Pten^{RNAi} (BDSC: 33643)

Figure 3.

Figure 3a.
w*;esg-Gal4^{NP7397},UAS-GFP,Tub-Gal80^{TS}/+;UAS-Notch^{RNAi} (VDRC: v27229)/+

Figure 3b.
w*;esg-Gal4^{NP7397},UAS-GFP,Tub-Gal80^{TS}/+;UAS-Notch^{RNAi} (VDRC: v27229)/+
w*;esg-Gal4^{NP7397},UAS-GFP,Tub-Gal80^{TS}/UAS-Notch^{RNAi} (VDRC: v100002);Ilp2^{KO} (BDSC: 30881)
w*;esg-Gal4^{NP7397},UAS-GFP,Tub-Gal80^{TS}/UAS-Notch^{RNAi} (VDRC: v100002);Ilp3^{KO} (BDSC: 30882)

Figure 3c.
w*;esg-Gal4^{NP7397},UAS-GFP,Tub-Gal80^{TS}/+;UAS-Notch^{RNAi} (VDRC: v27229)/+
w*;esg-Gal4^{NP7397},UAS-GFP,Tub-Gal80^{TS}/+;UAS-Notch^{RNAi} (VDRC: v27229)/UAS-Ilp2 (BDSC: 80936)
w*;esg-Gal4^{NP7397},UAS-GFP,Tub-Gal80^{TS}/+;UAS-Notch^{RNAi} (VDRC: v27229)/UAS-Ilp3

Figure 3d.
w*; StenExSX-4, Tub-Gal80^{TS}, LexAop-mCD8::GFP/+;LexAop-Notch^{shRNA}/+
w*; StenExSX-4, Tub-Gal80^{TS}, LexAop-mCD8::GFP/UAS-Ilp3^{RNAi} (VDRC: v106512);LexAop-Notch^{shRNA}/Mef2-Gal4 (BDSC: 27390)

Figure 4.

Figure 4b.
w*
w*;Ilp3>traF/+

Figure 4c.
w*
w*;trh>traF/+

Figure 4d.
w*
w*;Cg>traF/+

Figure 4e.
w*
w*;Glia>traF/+

Figure 4f.
w*
w*;nSyb>traF/+

Figure 4g.
w*;yolk-Gal4 (BDSC: 58814),UAS-Stinger (BDSC: 84277)/+
w*;yolk-Gal4 (BDSC: 58814),UAS-Stinger (BDSC: 84277)/+;Cg>traF/+
w*;;dsx^{M}-LexA,LexAop-mCD8::GFP (BDSC: 32203)/+
w*;;dsx^{M}-LexA,LexAop-mCD8::GFP (BDSC: 32203)/Cg>traF

Figure 4h.
w*;esg-Gal4^{NP7397},UAS-GFP,Tub-Gal80^{TS}/+;UAS-Notch^{RNAi} (VDRC: v27229)/+
w*;esg-Gal4^{NP7397},UAS-GFP,Tub-Gal80^{TS}/Ilp3>traF;UAS-Notch^{RNAi} (VDRC: v27229)/+
w*;esg-Gal4^{NP7397},UAS-GFP,Tub-Gal80^{TS}/+;UAS-Notch^{RNAi} (VDRC: v27229)/trh>traF
w*;esg-Gal4^{NP7397},UAS-GFP,Tub-Gal80^{TS}/+;UAS-Notch^{RNAi} (VDRC: v27229)/Cg>traF
w*;esg-Gal4^{NP7397},UAS-GFP,Tub-Gal80^{TS}/+;UAS-Notch^{RNAi} (VDRC: v27229)/Glia>traF
w*;esg-Gal4^{NP7397},UAS-GFP,Tub-Gal80^{TS}/+;UAS-Notch^{RNAi} (VDRC: v27229)/nSyb>traF

Figure 4i.
w*
w*;nSyb>traF/+
w*;R15A01-p65.AD (BDSC: 68837)/+;R71G01-Gal4.DBD (BDSC: 69507)/+
w*;R15A01-p65.AD (BDSC: 68837)/+;R71G01-Gal4.DBD (BDSC: 69507)/nSyb>traF,UAS-nRFP

Figure 5.
Figure 5a.
w*;yolk-Gal4 (BDSC: 58814),UAS-Stinger (BDSC: 84277)/+
w*;yolk-Gal4 (BDSC: 58814),UAS-Stinger (BDSC: 84277)/+;dsx>traF/+

Figure 5b.
w*;esg-Gal4^{NP7397},UAS-GFP,Tub-Gal80^{TS}/+;UAS-Notch^{RNAi} (VDRC: v27229)/+
w*;esg-Gal4^{NP7397},UAS-GFP,Tub-Gal80^{TS}/+;UAS-Notch^{RNAi} (VDRC: v27229)/dsx>traF
w*;esg-Gal4^{NP7397},UAS-GFP,Tub-Gal80^{TS}/+;UAS-Notch^{RNAi} (VDRC: v27229)/fru^{P1}>traF
w*;esg-Gal4^{NP7397},UAS-GFP,Tub-Gal80^{TS}/LexAop-grim-rpr;UAS-Notch^{RNAi} (VDRC: v27229)/fru^{P1}-LexA (BDSC: 66698)
w*;esg-Gal4^{NP7397},UAS-GFP,Tub-Gal80^{TS}/+;UAS-Notch^{RNAi} (VDRC: v27229)/fru^{P1}>traF,UAS-InR^{RNAi} (VDRC: v992)
w*;esg-Gal4^{NP7397},UAS-GFP,Tub-Gal80^{TS}/+;UAS-Notch^{RNAi} (VDRC: v27229)/fru^{P1}>traF,UAS-Akt$^{RNAi\#2}$ (VDRC: v2902)
w*;esg-Gal4^{NP7397},UAS-GFP,Tub-Gal80^{TS}/+;UAS-Notch^{RNAi} (VDRC: v27229)/fru^{P1}>traF,UAS-Akt$^{RNAi\#3}$ (NIG-Fly: 4006R-3)

Figure 5c.
w*;R15A01-p65.AD (BDSC: 68837)/+;R71G01-Gal4.DBD (BDSC: 69507)/UAS-mCD8::GFP (BDSC: 32185)
w*;R15A01-p65.AD (BDSC: 68837)/+;R71G01-Gal4.DBD (BDSC: 69507)/fru^{P1}>traF,UAS-mCD8::GFP (BDSC: 32185)

Figure 5d.
w*;esg-Gal4^{NP7397},UAS-GFP,Tub-Gal80^{TS}/+;UAS-Notch^{RNAi} (VDRC: v27229)/fru^{P1}-LexA (BDSC: 66698)
w*;esg-Gal4^{NP7397},UAS-GFP,Tub-Gal80^{TS}/LexAop-TrpA1;UAS-Notch^{RNAi} (VDRC: v27229)/fru^{P1}-LexA (BDSC: 66698)

Figure 5e.
w*;esg-Gal4^{NP7397},UAS-GFP,Tub-Gal80^{TS}/+;UAS-Notch^{RNAi} (VDRC: v27229)/+
w*;esg-Gal4^{NP7397},UAS-GFP,Tub-Gal80^{TS}/UAS-Notch^{RNAi} (VDRC: v100002);dsx^{1} (BDSC: 1679)/dsx$^{\Delta}$
w*;esg-Gal4^{NP7397},UAS-GFP,Tub-Gal80^{TS}/UAS-Notch^{RNAi} (VDRC: v100002);fru^{F} (BDSC: 66873)/Df(3R)fru$^{4-40}$ (BDSC: 66692)

Figure 5f.
w*;esg-Gal4^{NP7397},UAS-GFP,Tub-Gal80^{TS}/UAS-Notch^{RNAi} (VDRC: v100002)
w*;esg-Gal4^{NP7397},UAS-GFP,Tub-Gal80^{TS}/UAS-Notch^{RNAi} (VDRC: v100002);fru^{P1}-Gal4 (BDSC: 66696)/UAS-tra2^{RNAi} (BDSC: 28018)

Figure 5g.
w*;esg-Gal4^{NP7397},UAS-GFP,Tub-Gal80^{TS}/+;UAS-yki^{3SA} (BDSC: 28817)/+
w*;esg-Gal4^{NP7397},UAS-GFP,Tub-Gal80^{TS}/+;UAS-yki^{3SA} (BDSC: 28817)/fru^{P1}>traF
w*;esg-Gal4^{NP7397},UAS-GFP,Tub-Gal80^{TS}/+;UAS-yki^{3SA} (BDSC: 28817)/CG10116>traF

Figure 6.
Figure 6a.
w*;+/+;fru^{P1}-LexA (BDSC: 66698)/+
w*;;fru^{P1}>traF/+
w*;LexAop-grim-rp/+;fru^{P1}-LexA (BDSC: 66698)/+

Figure 6b.
w*;Ilp3-Gal4/+;UAS-Stinger (BDSC: 84278)/+
w*;Ilp3-Gal4/+;fru^{P1}>traF,UAS-Stinger (BDSC: 84278)/+

Figure 6c.
w*;;+/+
w*;;fru^{P1}>traF/+

Figure 6d.
w*;+/+;fru^{P1}-Gal4 (BDSC: 66696)/+
w*;UAS-rpr (BDSC: 5824)/+;fru^{P1}-Gal4 (BDSC: 66696)/+

Figure 6e.
w*;esg-LacZ^{k00606} (BDSC: 10359)/+;+/+
w*;esg-LacZ^{k00606} (BDSC: 10359)/+;fru^{P1}>traF/+

Figure 6f, g.
w*;;Ilp3^{KO}/+
w*;;Ilp3^{KO}/Ilp3^{KO}

Figure 6h.
w*;Ilp3-Gal4/+;nSyb-GAL80 (BDSC: 92154)/+
w*;Ilp3-Gal4/UAS-Ilp3^{RNAi} (VDRC: v106512);nSyb-GAL80 (BDSC: 92154)/+
w*;Ilp2-Gal4 (BDSC: 37516)/+
w*;Ilp2-Gal4 (BDSC: 37516)/UAS-Ilp3^{RNAi} (VDRC: v106512)

Supplementary figure 1.

Supplementary figure 1a.
w*;esg-Gal4^{NP7397},UAS-GFP,Tub-Gal80^{TS}/+;UAS-Notch^{RNAi} (VDRC: v27229)/+

Supplementary figure 1a.
w*;esg-Gal4^{NP7397},UAS-GFP,Tub-Gal80^{TS}/+;UAS-Notch^{RNAi} (VDRC: v27229)/+
w*;esg-Gal4^{NP7397},UAS-GFP,Tub-Gal80^{TS}/+;UAS-Notch^{RNAi} (VDRC: v27229)/UAS-sc^{RNAi} (BDSC: 41594)

Supplementary figure 2.

Supplementary figure 2a.
w*;esg-Gal4^{NP7397},UAS-GFP,Tub-Gal80^{TS}/+;UAS-Notch^{RNAi} (VDRC: v27229)/+
w*;esg-Gal4^{NP7397},UAS-GFP,Tub-Gal80^{TS}/+;UAS-Notch^{RNAi} (VDRC: v27229)/UAS-gce^{RNAi} (BDSC: 26323)
w*;esg-Gal4^{NP7397},UAS-GFP,Tub-Gal80^{TS}/+;UAS-Notch^{RNAi} (VDRC: v27229)/UAS-wit^{RNAi} (BDSC: 41906)
w*;esg-Gal4^{NP7397},UAS-GFP,Tub-Gal80^{TS}/+;UAS-Notch^{RNAi} (VDRC: v27229)/UAS-InR^{RNAi} (VDRC: v992)
w*;esg-Gal4^{NP7397},UAS-GFP,Tub-Gal80^{TS}/UAS-put^{RNAi} (BDSC: 35701);UAS-Notch^{RNAi} (VDRC: v27229)/+
w*;esg-Gal4^{NP7397},UAS-GFP,Tub-Gal80^{TS}/+;UAS-Notch^{RNAi} (VDRC: v27229)/UAS-babo^{RNAi} (BDSC: 41585)
w*;esg-Gal4^{NP7397},UAS-GFP,Tub-Gal80^{TS}/+;UAS-Notch^{RNAi} (VDRC: v27229)/UAS-CCAPR^{RNAi} (VDRC: v14767)
w*;esg-Gal4^{NP7397},UAS-GFP,Tub-Gal80^{TS}/UAS-SPR^{RNAi} (BDSC: 77389);UAS-Notch^{RNAi} (VDRC: v27229)/+
w*;esg-Gal4^{NP7397},UAS-GFP,Tub-Gal80^{TS}/+;UAS-Notch^{RNAi} (VDRC: v27229)/UAS-AstC-R2^{RNAi} (BDSC: 36888)
w*;esg-Gal4^{NP7397},UAS-GFP,Tub-Gal80^{TS}/+;UAS-Notch^{RNAi} (VDRC: v27229)/UAS-AstC-R1^{RNAi} (BDSC: 27506)
w*;esg-Gal4^{NP7397},UAS-GFP,Tub-Gal80^{TS}/+;UAS-Notch^{RNAi} (VDRC: v27229)/UAS-Met^{RNAi} (BDSC: 61935)
w*;esg-Gal4^{NP7397},UAS-GFP,Tub-Gal80^{TS}/+;UAS-Notch^{RNAi} (VDRC: v27229)/UAS-Proc-R^{RNAi} (BDSC: 29414)
w*;esg-Gal4^{NP7397},UAS-GFP,Tub-Gal80^{TS}/+;UAS-Notch^{RNAi} (VDRC: v27229)/UAS-TkR99D^{RNAi} (BDSC: 27513)
w*;esg-Gal4^{NP7397},UAS-GFP,Tub-Gal80^{TS}/+;UAS-Notch^{RNAi} (VDRC: v27229)/UAS-CCHa1-R^{RNAi} (BDSC: 27669)
w*;esg-Gal4^{NP7397},UAS-GFP,Tub-Gal80^{TS}/+;UAS-Notch^{RNAi} (VDRC: v27229/UAS-NPFR^{RNAi} (BDSC: 25939)
w*;esg-Gal4^{NP7397},UAS-GFP,Tub-Gal80^{TS}/+;UAS-Notch^{RNAi} (VDRC: v27229)/UAS-sNPFR^{RNAi} (BDSC: 27507)
w*;esg-Gal4^{NP7397},UAS-GFP,Tub-Gal80^{TS}/+;UAS-Notch^{RNAi} (VDRC: v27229)/UAS-Dh31-R^{RNAi} (BDSC: 25925)
w*;esg-Gal4^{NP7397},UAS-GFP,Tub-Gal80^{TS}/+;UAS-Notch^{RNAi} (VDRC: v27229)/UAS-Gyc76C^{RNAi} (BDSC: 57315)
w*;esg-Gal4^{NP7397},UAS-GFP,Tub-Gal80^{TS}/+;UAS-Notch^{RNAi} (VDRC: v27229)/UAS-CrzR^{RNAi} (BDSC: 26017)
w*;esg-Gal4^{NP7397},UAS-GFP,Tub-Gal80^{TS}/+;UAS-Notch^{RNAi} (VDRC: v27229)/UAS-CapaR^{RNAi} (BDSC: 27275)
w*;esg-Gal4^{NP7397},UAS-GFP,Tub-Gal80^{TS}/+;UAS-Notch^{RNAi} (VDRC: v27229)/UAS-PK2-R2^{RNAi} (BDSC: 36827)
w*;esg-Gal4^{NP7397},UAS-GFP,Tub-Gal80^{TS}/+;UAS-Notch^{RNAi} (VDRC: v27229)/UAS-PK2-R1^{RNAi} (BDSC: 29624)
w*;esg-Gal4^{NP7397},UAS-GFP,Tub-Gal80^{TS}/UAS-MsR2^{RNAi} (BDSC: 67869);UAS-Notch^{RNAi} (VDRC: v27229)/+
w*;esg-Gal4^{NP7397},UAS-GFP,Tub-Gal80^{TS}/+;UAS-Notch^{RNAi} (VDRC: v27229)/UAS-MsR1^{RNAi} (BDSC: 27529)
w*;esg-Gal4^{NP7397},UAS-GFP,Tub-Gal80^{TS}/+;UAS-Notch^{RNAi} (VDRC: v27229)/UAS-CCHa2-R^{RNAi} (BDSC: 25855)
w*;esg-Gal4^{NP7397},UAS-GFP,Tub-Gal80^{TS}/UAS-AstA-R2^{RNAi} (BDSC: 67864);UAS-Notch^{RNAi} (VDRC: v27229)/+
w*;esg-Gal4^{NP7397},UAS-GFP,Tub-Gal80^{TS}/+;UAS-Notch^{RNAi} (VDRC: v27229)/UAS-AstA-R1^{RNAi} (BDSC: 27280)
w*;esg-Gal4^{NP7397},UAS-GFP,Tub-Gal80^{TS}/+;UAS-Notch^{RNAi} (VDRC: v27229)/UAS-TkR86C^{RNAi} (BDSC: 31884)
w*;esg-Gal4^{NP7397},UAS-GFP,Tub-Gal80^{TS}/+;UAS-Notch^{RNAi} (VDRC: v27229)/UAS-FMRFaR^{RNAi} (BDSC: 25858)
w*;esg-Gal4^{NP7397},UAS-GFP,Tub-Gal80^{TS}/UAS-Pdfr^{RNAi} (BDSC: 42508);UAS-Notch^{RNAi} (VDRC: v27229)/+
w*;esg-Gal4^{NP7397},UAS-GFP,Tub-Gal80^{TS}/UAS-SIFaR^{RNAi} (BDSC: 44068);UAS-Notch^{RNAi} (VDRC: v27229)/+
w*;esg-Gal4^{NP7397},UAS-GFP,Tub-Gal80^{TS}/UAS-AkhR^{RNAi} (BDSC: 51710) ;UAS-Notch^{RNAi} (VDRC: v27229)/+
w*;esg-Gal4^{NP7397},UAS-GFP,Tub-Gal80^{TS}/+;UAS-Notch^{RNAi} (VDRC: v27229)/UAS-Lkr^{RNAi} (BDSC: 65934)

Supplementary figure 2b.

w*;esg-Gal4^{NP7397},UAS-GFP,Tub-Gal80^{TS}/+;UAS-Notch^{RNAi} (VDRC: v27229)/+
w*,UAS-PI3K92E^{CAAX} (BDSC: 8294);esg-Gal4^{NP7397},UAS-GFP,Tub-Gal80^{TS}/+;UAS-Notch^{RNAi} (VDRC: v27229)/+
w*;esg-Gal4^{NP7397},UAS-GFP,Tub-Gal80^{TS}/+;UAS-Notch^{RNAi} (VDRC: v27229)/UAS-Akt$^{RNAi\#2}$ (VDRC: v2902)
w*;esg-Gal4^{NP7397},UAS-GFP,Tub-Gal80^{TS}/+;UAS-Notch^{RNAi} (VDRC: v27229)/UAS-Akt$^{RNAi\#3}$ (NIG-Fly: 4006R-3)

Supplementary figure 3.

Supplementary figure 3a.

w*;esgGal4^{NP7397},UAS-GFP,Tub-Gal80^{TS}/+;Df(3L)$^{st-j7}$/+
w*;esgGal4^{NP7397},UAS-GFP,Tub-Gal80^{TS}/+;Df(3L)$^{st-j7}$/tra^{KO}

Supplementary figure 3b.

w*;esg-Gal4^{NP7397},UAS-GFP,Tub-Gal80^{TS}/+;UAS-Notch^{RNAi} (VDRC: v27229)/+
w*;esg-Gal4^{NP7397},UAS-GFP,Tub-Gal80^{TS}/UAS-Notch^{RNAi} (VDRC: v100002);Ilp5^{KO} (BDSC: 30884)

Supplementary figure 4.

Supplementary figure 4a.

w*;;+/+
w*;;Cg>traF/+

Supplementary figure 4b.

w*;;dsx^{M}-LexA,LexAop-mCD8::GFP (BDSC: 32203)/+
w*;;dsx^{M}-LexA,LexAop-mCD8::GFP (BDSC: 32203)/Cg>traF

Supplementary figure 4c.

w*;;UAS-mCD8::GFP (BDSC: 32185)/GMR14D03-Gal4 (BDSC: 47463)

Supplementary figure 4d.

w*;;+/+
w*;;nSyb>traF/+

Supplementary figure 4e.

w*;esg-Gal4^{NP7397},UAS-GFP,Tub-Gal80^{TS}/+;UAS-Notch^{RNAi} (VDRC: v27229)/+
w*;esg-Gal4^{NP7397},UAS-GFP,Tub-Gal80^{TS}/+;UAS-Notch^{RNAi} (VDRC: v27229)/Ilp2>traF
w*;esg-Gal4^{NP7397},UAS-GFP,Tub-Gal80^{TS}/+;UAS-Notch^{RNAi} (VDRC: v27229)/Akh>traF
w*;esg-Gal4^{NP7397},UAS-GFP,Tub-Gal80^{TS}/+;UAS-Notch^{RNAi} (VDRC: v27229)/Dh44>traF
w*;esg-Gal4^{NP7397},UAS-GFP,Tub-Gal80^{TS}/+;UAS-Notch^{RNAi} (VDRC: v27229)/Ilp7>traF
w*;esg-Gal4^{NP7397},UAS-GFP,Tub-Gal80^{TS}/+;UAS-Notch^{RNAi} (VDRC: v27229)/Pdf>traF
w*;esg-Gal4^{NP7397},UAS-GFP,Tub-Gal80^{TS}/+;UAS-Notch^{RNAi} (VDRC: v27229)/ARCEN>traF
w*;esg-Gal4^{NP7397},UAS-GFP,Tub-Gal80^{TS}/+;UAS-Notch^{RNAi} (VDRC: v27229)/CCAP>traF
w*;esg-Gal4^{NP7397},UAS-GFP,Tub-Gal80^{TS}/+;UAS-Notch^{RNAi} (VDRC: v27229)/R76F12>traF
w*;esg-Gal4^{NP7397},UAS-GFP,Tub-Gal80^{TS}/+;UAS-Notch^{RNAi} (VDRC: v27229)/R37F03>traF
w*;esg-Gal4^{NP7397},UAS-GFP,Tub-Gal80^{TS}/+;UAS-Notch^{RNAi} (VDRC: v27229)/R22C05>traF
w*;esg-Gal4^{NP7397},UAS-GFP,Tub-Gal80^{TS}/+;UAS-Notch^{RNAi} (VDRC: v27229)/R25F07>traF

Supplementary figure 5.

Supplementary figure 5b.

w*;;+/+
w*;;fru^{P1}>traF/+

Supplementary figure 5c.

w*;;fru^{V5}/+
w*;;fru^{V5}/fru^{P1}>traF
w*;;GMR19B09-Gal4 (BDSC: 48840)/UAS-mCD8::GFP (BDSC: 32185)
w*;; GMR19B09-Gal4 (BDSC: 48840)/fru^{P1}>traF,UAS-mCD8::GFP (BDSC: 32185)

Supplementary figure 5d and 5e.

w*;;+/+
w*;;fru^{P1}>traF/+

Supplementary figure 5f.
w*;esg-Gal4^{NP7397},UAS-GFP,Tub-Gal80^{TS}/+;UAS-ovo$^{svb.ACT}$/+

Supplementary figure 5g.
w*;esg-Gal4^{NP7397},UAS-GFP,Tub-Gal80^{TS}/+;UAS-Notch^{RNAi} (VDRC: v27229)/+
w*;esg-Gal4^{NP7397},UAS-GFP,Tub-Gal80^{TS}/+;UAS-Notch^{RNAi} (VDRC: v27229)/fru^{P1}>traF

Supplementary figure 6.

Supplementary figure 6a.
w*;;UAS-mCD8::GFP (BDSC: 32186)/fru^{P1}-Gal4 (BDSC: 66696)

Supplementary figure 6b, 6c.
w*;;+/+

Supplementary figure 6d.
w*;Ilp3-Gal4/+;UAS-Stinger (BDSC: 84278)/+

Supplementary figure 6e.
w*;;+/+
w*;;CG10116>traF/+

Supplementary figure 6f.
w*;;GMR94E02-Gal4 (BDSC: 40684)/UAS-mCD8::GFP (BDSC: 32185)
w*;;GMR94E02-Gal4 (BDSC: 40684)/fru^{P1}>traF,UAS-mCD8::GFP (BDSC: 32185)
w*;;GMR46B08-Gal4 (BDSC: 47361)/UAS-mCD8::GFP (BDSC: 32185)
w*;;GMR46B08-Gal4 (BDSC: 47361)/fru^{P1}>traF,UAS-mCD8::GFP (BDSC: 32185)
w*;;GMR69H10-Gal4 (BDSC: 46626)/UAS-mCD8::GFP (BDSC: 32185)
w*;;GMR69H10-Gal4 (BDSC: 46626)/fru^{P1}>traF,UAS-mCD8::GFP (BDSC: 32185)
w*;;GMR18B03-Gal4 (BDSC: 48798) /UAS-mCD8::GFP (BDSC: 32185)
w*;;GMR18B03-Gal4 (BDSC: 48798) /fru^{P1}>traF,UAS-mCD8::GFP (BDSC: 32185)

Supplementary figure 6g, 6h.
w*;;+/+
w*;;fru^{P1}>traF/+

Supplementary figure 6i.
w*;UAS-Stinger (BDSC: 84277)/Ilp3-Gal4
w*;UAS-Stinger (BDSC: 84277)/Ilp3-Gal4;nSyb-GAL80 (BDSC: 92154)/+
w*;Ilp3-Gal4/+;nSyb-GAL80 (BDSC: 92154)/+
w*;Ilp3-Gal4/UAS-Ilp3^{RNAi} (VDRC: v106512);nSyb-GAL80 (BDSC: 92154)/+
w*;Ilp2-Gal4 (BDSC: 37516)/+
w*;Ilp2-Gal4 (BDSC: 37516)/UAS-Ilp3^{RNAi} (VDRC: v106512)

Supplementary figure 6j.
w*;Ilp3-Gal4/+
w*;Ilp3-Gal4/UAS-Ilp3^{RNAi} (VDRC: v106512)

Supplementary figure 6k.
w*;UAS-brp.GFP (BDSC: 36291)/+;fru^{P1}-Gal4 (BDSC: 66696)/+
