# Supplementary material for: Sex of central neurons controls intestinal niche signalling to drive sex differences in stem cell activity and cancer susceptibility
Source: Nat Commun. 2026 Aug 22;17:10043. doi: 10.1038/s41467-026-76998-z (PMC13594308; doi:10.1038/s41467-026-76998-z)
Supplement: Supplementary file 2 [file 41467_2026_76998_MOESM2_ESM.pdf]

nature portfolio

Peer Review File

Sex of central neurons controls intestinal niche signalling to drive sex differences in stem cell activity and cancer susceptibility

Corresponding Author: Dr Bruno Hudry

This file contains all reviewer reports in order by version, followed by all author rebuttals in order by version.

Version 0:

Reviewer comments:

Reviewer #1

(Remarks to the Author)
The revised manuscript by Clot, Delanue et al., contains significant evidence that addresses the main comments I made in about original submission and strengthens the case for Fru expressing neurons regulating sexual dimorphism of intestinal tumours. Most significantly, the validation of their tissue specific tools for feminisation of intestinal niche compartment and systemic tissues and the expansion of their phenotype towards various tumour models. Congratulations on very compelling body of work.

Reviewer #2

(Remarks to the Author)
I was already positive about this manuscript, pending some "softening" of the authors' conclusions. I am satisfied with the authors response and feel the work is ready for publication in Nature Comm.

Reviewer #3

(Remarks to the Author)
Clot et al have done a fabulous job of addressing my concerns. The paper provides a very strong case for the sex of Fru1 neurons required for the sex difference in drosophila colon cancer. The study has multiple elements of novelty and strength. I have only 2 minor comments.

1. As stated in the response to my review, “Rather, the strength of our approach lies in its use as a genetically tractable system to uncover conserved principles and identify previously unrecognised pathways that may be relevant across diverse cancer contexts.”

I agree that drosophila is a powerful model. I’m less supportive of the notion of conserved principles and pathways in this case as the fly model of colon cancer involves silencing of notch rather than the notch activation observed in human CRC, and tumorigenesis in flies has the opposite sex bias in incidence as in humans. So, while the novelty and rigor of the study will make it of interest broadly, can the introduction and discussion do more to clarify the distinctions between flies and humans with regard to conserved principles and pathways? In my view, the inclusion of human studies in the introduction raises an expectation that the research is more directly to human disease.

2. Y chromosomes are often lost in cancers in aging males (bladder, heme malignancies) highlighting that the Y chromosome can have tumor suppressor as well as oncogene functions. Thus, it is difficult to simply ascribe Y chromosome effects to greater cancer risk and decreased survival in male cancer patients. Two X chromosomes are more consistsnetly tumor suppressive and therefore more likely to contribute to sex differences in cancer incidence. I appreciate that the authors have added comments about the X chromosome, but I think the introduction should do more to detail the mixed Y chromosome effects and more consistent X chromosome effects on cancer risk.

Open Access This Peer Review File is licensed under a Creative Commons Attribution 4.0 International License, which permits use, sharing, adaptation, distribution and reproduction in any medium or format, as long as you give appropriate credit to the original author(s) and the source, provide a link to the Creative Commons license, and indicate if changes were made.
In cases where reviewers are anonymous, credit should be given to 'Anonymous Referee' and the source.
The images or other third party material in this Peer Review File are included in the article's Creative Commons license, unless indicated otherwise in a credit line to the material. If material is not included in the article's Creative Commons license and your intended use is not permitted by statutory regulation or exceeds the permitted use, you will need to obtain permission directly from the copyright holder.
To view a copy of this license, visit https://creativecommons.org/licenses/by/4.0/

Response to Reviewers

We thank the Reviewers for their time examining our manuscript and providing valuable comments. We have followed all their suggestions. We are providing a point-by-point response to their specific points below. Changes in the manuscript are highlighted in red.

Reviewer #1 (Remarks to the Author):
The revised manuscript by Clot, Delanoue et al., contains significant evidence that addresses the main comments I made in about original submission and strengthens the case for Fru expressing neurons regulating sexual dimorphism of intestinal tumours. Most significantly, the validation of their tissue specific tools for feminisation of intestinal niche compartment and systemic tissues and the expansion of their phenotype towards various tumour models. Congratulations on very compelling body of work.
We want to thank the Reviewer for the positive assessment of our work.

Reviewer #2 (Remarks to the Author):
I was already positive about this manuscript, pending some "softening" of the authors' conclusions. I am satisfied with the authors response and feel the work is ready for publication in Nature Comm.
We want to thank the Reviewer for the positive assessment of our work.

Reviewer #3 (Remarks to the Author):
Clot et al have done a fabulous job of addressing my concerns. The paper provides a very strong case for the sex of Fru1 neurons required for the sex difference in drosophila colon cancer. The study has multiple elements of novelty and strength.
We want to thank the Reviewer for the positive assessment of our work.

I have only 2 minor comments.
1. As stated in the response to my review, "Rather, the strength of our approach lies in its use as a genetically tractable system to uncover conserved principles and identify previously unrecognised pathways that may be relevant across diverse cancer contexts."
I agree that drosophila is a powerful model. I'm less supportive of the notion of conserved principles and pathways in this case as the fly model of colon cancer involves silencing of notch rather than the notch activation observed in human CRC, and tumorigenesis in flies has the opposite sex bias in incidence as in humans. So, while the novelty and rigor of the study will make it of interest broadly, can the introduction and discussion do more to clarify the distinctions between flies and humans with regard to conserved principles and pathways? In my view, the inclusion of human studies in the introduction raises an expectation that the research is more directly to human disease.
We agree that this point was insufficiently clear. By "conserved principles and pathways," we refer to the two main findings of this manuscript: the role of the intrinsic sexual identity of central neurons in the brain–gut axis in sex-biased tumorigenesis, and the role of a locally secreted growth factor, here Ilp3, as a downstream effector. The Akt/Insulin pathway is also highly conserved and plays a key role in tumour growth across species. We fully agree that Drosophila and human biology, as well as the specific features of each organ system, are sufficiently distinct that our findings cannot be directly extrapolated to a specific human disease context. This may explain why different tumour suppressors are involved and why sex biases can differ between species. Nevertheless, the general principle that neuronal sexual identity can influence peripheral tumour niches may represent a conserved and previously unrecognized mechanism contributing to sex differences in tumour development across species. We have clarified this point by adding new sentences to the Discussion (please see text **lines 470-475**).

2. Y chromosomes are often lost in cancers in aging males (bladder, heme malignancies) highlighting that the Y chromosome can have tumor suppressor as well as oncogene functions. Thus, it is difficult to simply ascribe Y chromosome effects to greater cancer risk and decreased survival in male cancer patients. Two X chromosomes are more consistsnetly tumor suppressive and therefore more likely to contribute to sex differences in cancer incidence. I appreciate that the authors have added comments about the X chromosome, but I think the introduction should do more to detail the mixed Y chromosome effects and more consistent X chromosome effects on cancer risk.
We agree with the Reviewer and have revised the manuscript to clarify this point (please see the new text in the Introduction, **lines 64–67**).
