# Supplementary material for: Sex of central neurons controls intestinal niche signalling to drive sex differences in stem cell activity and cancer susceptibility
Source: Nat Commun. 2026 Aug 22;17:10043. doi: 10.1038/s41467-026-76998-z (PMC13594308; doi:10.1038/s41467-026-76998-z)
Supplement: Supplementary file 3 [file 41467_2026_76998_MOESM3_ESM.pdf]

nature portfolio

Corresponding author(s): Bruno Hudry

Last updated by author(s): Jul 25, 2026

Reporting Summary

Nature Portfolio wishes to improve the reproducibility of the work that we publish. This form provides structure for consistency and transparency in reporting. For further information on Nature Portfolio policies, see our Editorial Policies and the Editorial Policy Checklist.

Statistics

For all statistical analyses, confirm that the following items are present in the figure legend, table legend, main text, or Methods section.

n/a	Confirmed	
☐	☒	The exact sample size (*n*) for each experimental group/condition, given as a discrete number and unit of measurement
☐	☒	A statement on whether measurements were taken from distinct samples or whether the same sample was measured repeatedly
☐	☒	The statistical test(s) used AND whether they are one- or two-sided<br>*Only common tests should be described solely by name; describe more complex techniques in the Methods section.*
☒	☐	A description of all covariates tested
☐	☒	A description of any assumptions or corrections, such as tests of normality and adjustment for multiple comparisons
☐	☒	A full description of the statistical parameters including central tendency (e.g. means) or other basic estimates (e.g. regression coefficient) AND variation (e.g. standard deviation) or associated estimates of uncertainty (e.g. confidence intervals)
☐	☒	For null hypothesis testing, the test statistic (e.g. *F*, *t*, *r*) with confidence intervals, effect sizes, degrees of freedom and *P* value noted<br>*Give P values as exact values whenever suitable.*
☒	☐	For Bayesian analysis, information on the choice of priors and Markov chain Monte Carlo settings
☒	☐	For hierarchical and complex designs, identification of the appropriate level for tests and full reporting of outcomes
☒	☐	Estimates of effect sizes (e.g. Cohen's *d*, Pearson's *r*), indicating how they were calculated

Our web collection on statistics for biologists contains articles on many of the points above.

Software and code

Policy information about availability of computer code

Data collection	No software was used for data collection.
Data analysis	All statistical analyses were carried out using ImageJ (2015), Microsoft Excel (version 16.16.27), and GraphPad Prism 9.2.0 (283).

For manuscripts utilizing custom algorithms or software that are central to the research but not yet described in published literature, software must be made available to editors and reviewers. We strongly encourage code deposition in a community repository (e.g. GitHub). See the Nature Portfolio guidelines for submitting code & software for further information.

Data

Policy information about availability of data

All manuscripts must include a data availability statement. This statement should provide the following information, where applicable:

- Accession codes, unique identifiers, or web links for publicly available datasets
- A description of any restrictions on data availability
- For clinical datasets or third party data, please ensure that the statement adheres to our policy

All data is available in the main text or the supplementary data. Materials generated for the study are available from the corresponding authors on request.

Research involving human participants, their data, or biological material

Policy information about studies with human participants or human data. See also policy information about sex, gender (identity/presentation), and sexual orientation and race, ethnicity and racism.

Reporting on sex and gender	N/A
Reporting on race, ethnicity, or other socially relevant groupings	N/A
Population characteristics	N/A
Recruitment	N/A
Ethics oversight	N/A

Note that full information on the approval of the study protocol must also be provided in the manuscript.

Field-specific reporting

Please select the one below that is the best fit for your research. If you are not sure, read the appropriate sections before making your selection.

[x] Life sciences [ ] Behavioural & social sciences [ ] Ecological, evolutionary & environmental sciences

For a reference copy of the document with all sections, see nature.com/documents/nr-reporting-summary-flat.pdf

Life sciences study design

All studies must disclose on these points even when the disclosure is negative.

Sample size	No a priori sample-size calculation was performed, sample size was set according to the reproducibility of each experiment. ARRIVE guidelines had been followed for the study and the maximum number of replicates were used for each experiment above which additional replicates did not alter the statistical significance.
Data exclusions	No data were excluded from the analyses.
Replication	Each unique experiments were repeated at least three independent times (n). "n" refers to the number of biological replicates for each experimental groups. The number of technical replicates, the experimental units, and number of experimental units allocated to each group are indicated for all experiments in the Figures and/or in the Figure legends. All attempts at replication were successful.
Randomization	Samples of each specific genotype were randomly selected for analysis.
Blinding	All experiments were conducted single-blind. Each experimental group were given numbers prior to dissection and analysis. Only after data were recorded experimental numbers were brought with the genotypes/treatment groups.

Reporting for specific materials, systems and methods

We require information from authors about some types of materials, experimental systems and methods used in many studies. Here, indicate whether each material, system or method listed is relevant to your study. If you are not sure if a list item applies to your research, read the appropriate section before selecting a response.

Materials & experimental systems

n/a	Involved in the study	
[ ]	[x]	Antibodies
[x]	[ ]	Eukaryotic cell lines
[x]	[ ]	Palaeontology and archaeology
[ ]	[x]	Animals and other organisms
[x]	[ ]	Clinical data
[x]	[ ]	Dual use research of concern
[x]	[ ]	Plants

Methods

n/a	Involved in the study	
[x]	[ ]	ChIP-seq
[x]	[ ]	Flow cytometry
[x]	[ ]	MRI-based neuroimaging

Antibodies

Antibodies used	Rabbit anti-phospho-histone H3 Ser10 (9701L, Cell Signalling Technology); RRID: AB_331535; validation: vendor suitable for W, IHC-P,

IF-IC
Chicken anti-GFP, 1/10 000 Abcam Cat#ab13970; RRID: AB_300798; validation: vendor suitable for WB, ICC/IF
Mouse anti-Prospero (1:10, DSHB MR1A); RRID: AB_528440; validation: vendor suitable for: WB, ICC/IF
Chicken anti-βGal (1:1,000, GeneTex GTX77365); RRID: AB_379834; validation: vendor suitable for: WB, ICC/IF
Rat anti-FruM (Clone #1C1, 1:10; gift from K. Sato, generated and validated in PMID: 35124238)
Rat anti-Ilp2 (1:400; generated and validated in PMID: 19723496)
Rabbit anti-V5-Tag (D3H8Q) (1:100, Ozyme); RRID: AB_2687461; validation: vendor suitable for: WB, ICC/IF

Validation

Rabbit anti-phospho-histone H3 Ser10 (9701L, Cell Signalling Technology); RRID: AB_331535; validation: vendor suitable for W, IHC-P, IF-IC
Chicken anti-GFP, 1/10 000 Abcam Cat#ab13970; RRID: AB_300798; validation: vendor suitable for WB, ICC/IF
Mouse anti-Prospero (1:10, DSHB MR1A); RRID: AB_528440; validation: vendor suitable for: WB, ICC/IF
Chicken anti-βGal (1:1,000, GeneTex GTX77365); RRID: AB_379834; validation: vendor suitable for: WB, ICC/IF
Rat anti-FruM (Clone #1C1, 1:10; gift from K. Sato, generated and validated in PMID: 35124238)
Rat anti-Ilp2 (1:400; generated and validated in PMID: 19723496)
Rabbit anti-V5-Tag (D3H8Q) (1:100, Ozyme); RRID: AB_2687461; validation: vendor suitable for: WB, ICC/IF

Animals and other research organisms

Policy information about studies involving animals; ARRIVE guidelines recommended for reporting animal research, and Sex and Gender in Research

Laboratory animals

Fly strains
Drivers: esg-Gal4NP7397, UAS-GFP, Tub-Gal80TS chromosome, dsxM-LexA (this study, see below for details), VT063223-p65.AD (BDSC: 75204, Flybase ID: FBti0190631), R10G10-p65.AD (BDSC: 70529, Flybase ID: FBst0070529), CG10116-Gal4.DBD (BDSC: 91399, Flybase ID: FBst0091399), fruP1-Gal4 (BDSC: 66696, Flybase ID: FBst0066696), fruP1-LexA (BDSC: 66698, Flybase ID: FBst0066698), yolk-Gal4 (BDSC: 58814, Flybase ID: FBst0058814), GMR19B09-Gal4 (BDSC: 48840, Flybase ID: FBst0048840), R15A01-p65.AD (BDSC: 68837, Flybase ID: FBst0068837), R71G01-Gal4.DBD (BDSC: 69507, Flybase ID: FBst0069507), Ilp3-Gal4 (gift from L. Erin O'Brien, Flybase ID: FBtp0069556), tubP-Gal80TS (BDSC: 7108, Flybase ID: FBst0007108), GMR94E02-Gal4 (BDSC: 40684, Flybase ID: FBst0040684), GMR46B08-Gal4 (BDSC: 47361, Flybase ID: FBst0047361), GMR69H10-Gal4 (BDSC: 46626, Flybase ID: FBst0046626), GMR18B03-Gal4 (BDSC: 48798, Flybase ID: FBst0048798), esgLacZk00606 (BDSC: 10359, Flybase ID: FBst0010359), Ilp2-Gal4 (BDSC: 37516, Flybase ID: FBst0037516), nSyb-GAL80 (BDSC: 92154, Flybase ID: FBst0092154), Mef2-Gal4 (BDSC: 27390, Flybase ID: FBti0115746), StenExSX-4, Tub-Gal80TS, LexAop-mCD8::GFP chromosome (gift from Y. Kwon, Flybase ID: FBti0185052), GMR14D03-Gal4 (BDSC: 47463, Flybase ID: FBti0133237).

UAS transgenes: UAS-traF (BDSC: 4590, Flybase ID: FBst0004590), UAS-InR (BDSC: 8262, Flybase ID: FBst0008262), UAS-Ilp3 (gift from H. Stocker, Flybase ID: FBal0141675), UAS-Ilp2 (BDSC: 80936, Flybase ID: FBst0080936), UAS-PI3K92ECAAX 581 (BDSC: 8294, Flybase ID: FBst0008294), UAS-mCD8::GFP (BDSC: 32185, Flybase ID: FBst0032185), UAS-mCD8::GFP (BDSC: 32186, Flybase ID: FBst0032186), UAS-Stinger (BDSC: 84277, Flybase ID: FBst0084277), UAS-Stinger (BDSC: 84278, Flybase ID: FBst0084278), LexAop-grim-rpr (giftfrom T. Lee, Flybase ID: FBtp0165761), LexAop-mCD8::GFP (BDSC: 32203, Flybase ID: FBst0032203), UAS-rpr (BDSC: 5824, Flybase ID: FBst0005824), UAS-brp.GFP (BDSC: 36291, Flybase ID: FBst0036291), LexAop-TrpA1 (Flybase ID: FBal0349259), UAS-yki3SA (BDSC: 28817, Flybase ID: FBti0127382), UAS-ovosvb.ACT (gift from C. Polesello, Flybase ID: FBtp0134164).

RNAi transgenes: UAS-InR RNAi (VDRC: v992, Flybase ID: FBst0471628), UAS-AkhR RNAi (BDSC: 51710, Flybase ID: FBst0051710), UAS-Akt RNAi#1 (BDSC: 33615, allele ID: FBst0033615), UAS-Pten RNAi (BDSC: 33643, Flybase ID: FBst0033643), UAS-gce RNAi (BDSC: 26323, Flybase ID: FBst0026323), UAS-wit RNAi (BDSC: 41906, Flybase ID: FBst0041906), UAS-put RNAi (BDSC: 35701, Flybase ID: FBst0035701), UAS-babo RNAi (BDSC: 41585, Flybase ID: FBst0041585), UAS-CCAPR RNAi (VDRC: v14767, Flybase ID: FBst0451587), UAS-SPR RNAi (BDSC: 77389, Flybase ID: FBst0077389), UAS-AstC-R2 RNAi (BDSC: 36888, Flybase ID: FBst0036888), UAS-AstCR1 RNAi (BDSC: 27506, Flybase ID: FBst0027506), UAS-Met RNAi (BDSC: 61935, Flybase ID: FBst0061935), UAS-Proc-R RNAi (BDSC: 29414, Flybase ID: FBst0029414), UAS-TkR99D RNAi (BDSC: 27513, Flybase ID: FBst0027513), UAS-CCHa1-R RNAi (BDSC: 27669, Flybase ID: FBst0027669), UAS-NPFR RNAi (BDSC: 25939, Flybase ID: FBst0025939), UAS-sNPFR RNAi (BDSC: 27507, Flybase ID: FBst0027507), UAS-Dh31-R RNAi (BDSC: 25925, Flybase ID: FBst0025925), UAS-Gyc76C RNAi (BDSC: 57315, Flybase ID: FBst0057315), UAS-Crz RNAi (BDSC: 26017, Flybase ID: FBst0026017), UAS-CapaR RNAi (BDSC: 27275, Flybase ID: FBst0027275), UAS-PK2-R2 RNAi (BDSC: 36827, Flybase ID: FBst0036827), UAS-PK2-R1 RNAi (BDSC: 29624, Flybase ID: FBst0029624), UAS-MsR2 RNAi (BDSC: 67869, Flybase ID: FBst0067869), UAS-MsR1 RNAi (BDSC: 27529, Flybase ID: FBst0027529), UAS-CCHa2-R RNAi (BDSC: 25855, Flybase ID: FBst0025855), UAS-AstA-R2 RNAi (BDSC: 67864, Flybase ID: FBst0067864), UAS-AstA-R1 RNAi (BDSC: 27280, Flybase ID: FBst0027280), UAS-TkR86C RNAi (BDSC: 31884, Flybase ID: FBst0031884), UAS-FMRFaR RNAi (BDSC: 25858, Flybase ID: FBst0025858), UAS-Pdfr RNAi (BDSC: 42508, Flybase ID: FBst0042508), UAS-SIFaR RNAi (BDSC: 44068, Flybase ID: FBst0044068), UAS-Lkr RNAi (BDSC: 65934, Flybase ID: FBst0065934), UAS-sc RNAi (BDSC: 41594, Flybase ID: FBst0041594), UAS-Ilp3 RNAi (VDRC: v106512, Flybase ID: FBst0478336), UAS-Notch RNAi (VDRC: v27229, Flybase ID: FBst0456826), UAS-Notch RNAi (VDRC: v100002, Flybase ID: FBst0471876), LexAop-Notch shRNA (this study, see below for details), UAS-Akt RNAi#2 (VDRC: v2902, Flybase ID: FBti0083396), UAS-Akt RNAi#3 (NIG-Fly: 4006R-3, Flybase ID: FBti0246817), UAS-tra2 RNAi (BDSC: 28018, FlyBase ID: 617 FBti0128004).

Mutants: Ilp3 KO (BDSC: 30882, Flybase ID: FBst0030882), Ilp2 KO (BDSC: 30881, Flybase ID: FBst0030881), Ilp5 KO (BDSC: 30884, Flybase ID: FBst0030884), dsx1 (BDSC: 1679, Flybase ID: FBst0001679), dsxΔ (Flybase ID: FBal0325111), fruV5 (gift from Y. Pan, Flybase ID: FBal0366865), fruF (BDSC: 66873, Flybase ID: FBst0066873), Df(3R)fru4-40 (BDSC: 66692, Flybase ID: FBst0066692).

Feminising alleles: The following femising transgenic alleles were generated in this study (see below for details): tra>traF, dsx>traF, fruP1>traF, Cg>traF, CG10116>traF, Ilp2>traF, Ilp7>traF, Dh44>traF, CCAP>traF, nSyb>traF, Pdf>traF, trh>traF, Akh>traF, R20C06>traF, R76F12>traF, R37F03>traF, R22C05>traF, R25F07>traF, Glia>traF, ARCEN>traF, and Ilp3>traF.

Animals were reared on fly food containing (per liter): 10g of agar, 82.5g polenta, 34g dry yeast and 3,75g Moldex (per liter, diluted in ethanol). All experimental flies were kept in incubators at 25°C, and on a 12hr light/dark cycle. Flies were transferred to fresh vials every 3 days, and fly density was kept to a maximum of 15 flies per vial.

Wild animals	No wild animals were used in the study.
Reporting on sex	Both sexes were included. Sex-based analyses were performed.
Field-collected samples	No field collected samples were used in the study
Ethics oversight	N/A

Note that full information on the approval of the study protocol must also be provided in the manuscript.

Plants

Seed stocks	N/A
Novel plant genotypes	N/A
Authentication	N/A
